# Dysregulation of amino acid metabolism upon rapid depletion of cap-binding protein eIF4E

**DOI:** 10.1101/2023.05.11.540079

**Published:** 2023-05-12

**Authors:** Paige D. Diamond, Nicholas J. McGlincy, Nicholas T. Ingolia

**Affiliations:** 1 Department of Molecular and Cell Biology, University of California, Berkeley; 2 Center for Computational Biology and California Institute for Quantitative Biosciences, University of California, Berkeley

## Abstract

Protein synthesis is a crucial but metabolically costly biological process that must be tightly coordinated with cellular needs and nutrient availability. In response to environmental stress, translation initiation is modulated to control protein output while meeting new demands. The cap-binding protein eIF4E—the earliest contact between mRNAs and the translation machinery—serves as one point of control, but its contributions to mRNA-specific translation regulation remain poorly understood. To survey eIF4E-dependent translational control, we acutely depleted eIF4E and determined how this impacts protein synthesis. Despite its essentiality, eIF4E depletion had surprisingly modest effects on cell growth and protein synthesis. Analysis of transcript-level changes revealed that long-lived transcripts were downregulated, likely reflecting accelerated turnover. Paradoxically, eIF4E depletion led to simultaneous upregulation of genes involved in catabolism of aromatic amino acids, which arose as secondary effects of reduced protein biosynthesis on amino acid pools, and genes involved in the biosynthesis of amino acids. These futile cycles of amino acid synthesis and degradation were driven, in part, by translational activation of *GCN4*, a transcription factor typically induced by amino acid starvation. Furthermore, we identified a novel regulatory mechanism governing translation of *PCL5*, a negative regulator of Gcn4, that provides a consistent protein-to-mRNA ratio under varied translation environments. This translational control was partial dependent on a uniquely long poly-(A) tract in the *PCL5* 5′ UTR and on poly-(A) binding protein. Collectively, these results highlight how eIF4E connects translation to amino acid homeostasis and stress responses and uncovers new mechanisms underlying how cells tightly control protein synthesis during environmental challenges.

## Introduction

Protein synthesis, a vital step in gene expression and a significant biosynthetic process, is tightly regulated in response to nutrient status and environmental cues (Crawford and Pavitt, 2019; Hershey et al., 2012; Liu and Qian, 2014; Sonenberg and Hinnebusch, 2009). Often, this regulation affects translation initiation, a rate-limiting step and a point of commitment to protein synthesis (Shah et al., 2013). In eukaryotes, deeply conserved pathways modulate overall protein synthesis levels by controlling recognition of the distinctive 5′ cap on mRNAs and recruitment of the initiator tRNA to begin translation. These pathways also control transcript-specific expression, and sequence features in mRNAs play important roles in determining levels of protein synthesis (Park et al., 2011; Sen et al., 2016; Thoreen et al., 2012; Zinshteyn et al., 2017). Inhibitory upstream open reading frames (uORFs) divert initiation machinery and ribosomes from the main ORF, thus inhibiting translation in a context-dependent manner that can be relieved in stress (Brar et al., 2012; Calkhoven et al., 2000; Lu et al., 2004; Young and Wek, 2016). Translation of *GCN4*, a master regulator of amino acid starvation response in yeast, is a prime example of such uORF-dependent translation (Hinnebusch, 2005; Mueller and Hinnebusch, 1986). In response to amino acid deprivation, the kinase Gcn2 phosphorylates the translation initiation factor eIF2α (Dever et al., 1992; Hinnebusch, 2005), inhibiting bulk translation by decreasing the availability of eIF2 complex with initiator tRNA. *GCN4*, along with other stress-responsive proteins, overcome this inhibition to ensure the production needed to respond to stress. However, the mechanisms by which these transcripts are differentially translated is not fully understood.

The cap-binding protein, eIF4E, makes the first contact between a transcript and the translation initiation machinery during a typical initiation event. By recognizing the 5′m^7^G cap, a hallmark feature of eukaryotic mRNAs, eIF4E plays a central role in the canonical, cap-dependent pathway for translation initiation (Marcotrigiano et al., 1997; Matsuo et al., 1997; von der Haar et al., 2004). It also regulates mRNA stability by preventing decapping and degradation, perhaps through steric hindrance of decapping machinery (Vilela, 2000). Differential binding of cap-binding proteins to specific mRNAs regulates translational responses to environmental stresses in mammalian systems. Transcripts with 5′-terminal oligopyrimidine (5′TOP) tracts, which encode ribosomal proteins and elongation factors, are particularly sensitive to growth conditions and amino acid availability (Fonseca et al., 2015; Meyuhas and Kahan, 2015). In starvation, La-related protein 1 (LARP1) binds the caps of 5′TOP mRNAs to impede access of eIF4E to these specific transcripts (Lahr et al., 2017; Philippe et al., 2020). While *Saccharomyces cerevisiae* lacks this mode of regulation, yeast eIF4E is encoded by *CDC33* and was first identified through its cell cycle arrest phenotype (Reid and Hartwell, 1977). The link between eIF4E and cell-cycle progression has been attributed to translational control of the G1 cyclin 3 (Cln3), which drives the transition from G1 to S phase (Danaie et al., 1999). Furthermore, differential engagement of eIF4E across yeast mRNAs has been reported both *in vitro* by smFRET and *in vivo* by RNA-immunoprecipitation sequencing (Çetin and O’Leary, 2022; Costello et al., 2015). These findings raise the broader question, whether eIF4E might preferentially promote translation of certain transcripts, based features of the mRNAs, to control functionally related genes.

To understand the role of eIF4E in translation regulation, we investigated its mRNA-specific effects by profiling changes in gene expression and cell physiology after rapid depletion of eIF4E protein. Depletion of eIF4E preferentially destabilized long-lived mRNAs and reduced bulk protein synthesis. These conditions strongly induced genes involved in the catabolism of aromatic amino acids as secondary effects of reducing protein biosynthesis and the resultant rebalancing of amino acid pools. Furthermore, reductions in eIF4E levels caused translational activation of *GCN4*, a key regulator of stress responsive translation that is typically induced by amino acid starvation. Interestingly, translation of *GCN4* occurred through a non-canonical mechanism instead of relying on the well-established effects of eIF2α phosphorylation. Additionally, we noted translational repression of *PCL5*, a negative regulator of Gcn4, in eIF4E depleted cells, which may have further contributed to imbalanced amino acid pools. This work provided new insights into the regulation of Gcn4 activity through Pcl5-mediated feedback control and highlight the role of yeast eIF4E in control of nutrient-responsive gene regulatory networks.

## Results

### eIF4E depletion destabilizes short, stable transcripts with few direct effects on translation

**Figure 1:**
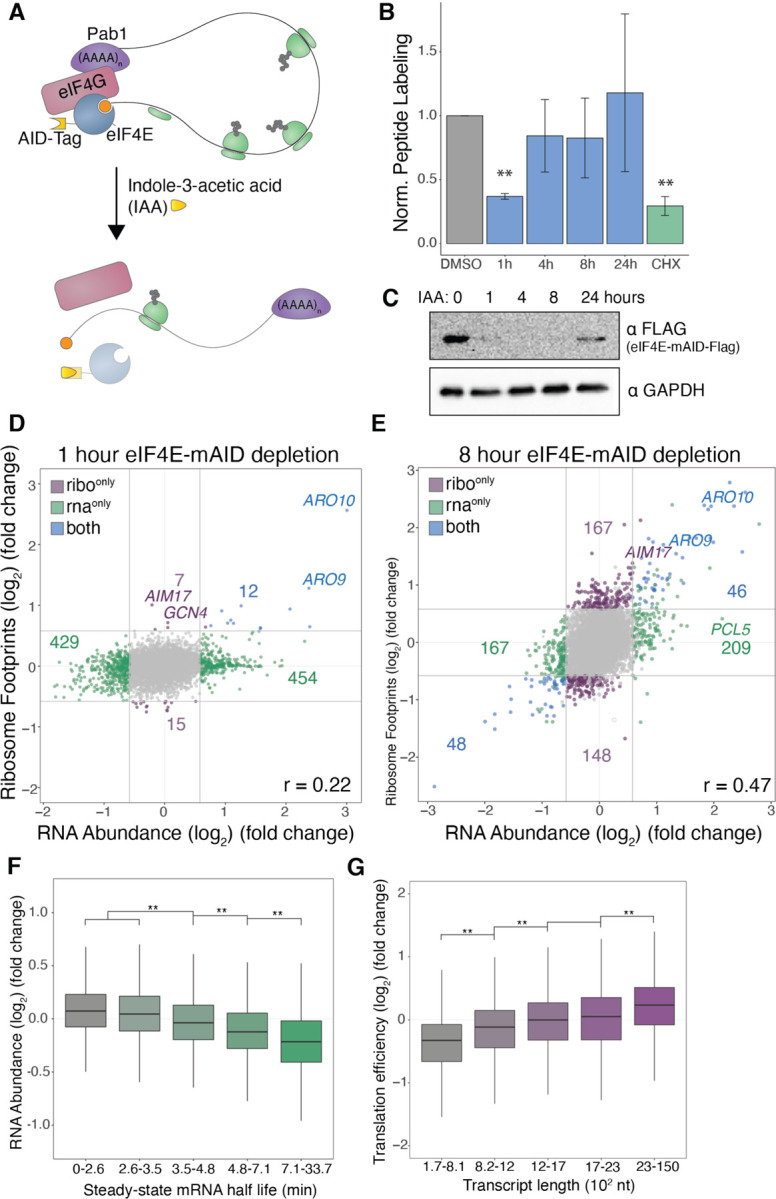
Transcript-level sensitivities to eIF4E depletion. **(A)** Schematic of auxin-inducible degron (AID) tagging and conditional depletion of eIF4E. **(B)** Bulk translation measured by nascent peptide metabolic labeling in eIF4E depleted cells. Indole-3-acetic acid (IAA) or cycloheximide (CHX) was added for indicated durations and maintained during a 2-hour labeling period with L-Homopropargylglycine (HPG). Median intensities of the Alexa Fluor^™^ 488 (HPG) signal normalized to that of DMSO treated cells. (**) represents p < 0.05 calculated by Student’s t test. Error bars represent standard error of the mean, n=2. **(C)** Western blot for eIF4E-mAID-Flag expression levels over the course of IAA depletion. **(D)** Differential expression after 1 hour of eIF4E depletion measured by RNA-seq and ribosome profiling. IAA-treated cells are compared with DMSO-treated controls. Color represents significant (adjusted p-value < 0.05) and substantial (absolute fold-change (log_2_) > 0.58) changes. Correlation coefficient (Pearson’s) calculated between log_2_ fold changes of RPF and RNA abundance. **(E)** Same as (D) except 8-hour IAA treatment. **(F)** Box plots of RNA abundance fold change (log_2_) for transcripts grouped based on steady-state mRNA half-life^45^. (**) indicates p < 0.05; one-way ANOVA test followed by Tukey’s HSD test. **(G)** Box plots of translation efficiency fold change (log_2_) for transcripts grouped based on length. (**) indicates p < 0.05; one-way ANOVA test followed by Tukey’s HSD test.

To investigate the contribution of the cap-binding protein eIF4E to the specificity of translation initiation across the transcriptome, we acutely depleted eIF4E (encoded by *CDC33* in *S. cerevisiae*) and measured the translational consequences. Prior studies relied on temperature-sensitive alleles, which require shifts to non-permissive temperatures that inhibit translation initiation even in wild-type cells and may have complex and incomplete molecular effects (Groušl et al., 2009). To avoid complications associated with temperature sensitivity, we depleted eIF4E using the auxin-inducible degron (AID) system, which allows conditional depletion of a target protein via degradation by the ubiquitin-proteasome system (Kubota et al., 2013; Nishimura et al., 2009) (Figure 1A), Degradation of eIF4E was rapidly induced by adding the auxin indole-3-acetic acid (IAA), and <10% of protein remained after 60 minutes (Figure 1C, S1G). To measure changes to bulk protein synthesis levels following eIF4E depletion, we carried out metabolic labeling of nascent peptides through addition of methionine analog, L-homopropargylglycine (HPG) for 120 minutes (Wiltschi et al., 2008) (Figure 1B, 1A–F). We then measured nascent peptide labeling by flow cytometry, after a fluorophore was added to incorporated HPG using click chemistry. We observed an approximate 50% reduction in nascent peptide labeling after 1 hour IAA treatment relative to DMSO treated cells (Figure 1B). This reduction in bulk protein synthesis was also supported by polysome profiling after 1 hour of IAA treatment (Figure S1H), which showed an increased proportion of free ribosomal subunits and monosomes and a depletion of polysomes, indicative of a reduction in translation initiation.

To determine the impact of eIF4E depletion globally on mRNA stability and protein synthesis, we measured changes in mRNA abundance and translation using RNA-sequencing and ribosome profiling (Ingolia et al., 2009). By comparing cells treated with IAA for 1 hour to DMSO control cells, we hoped to capture the most immediate gene expression changes attributed to eIF4E depletion, rather than the accumulation of secondary effects. We observed widespread changes in the transcriptome following eIF4E depletion; 454 transcripts were significantly upregulated and 429 transcripts were downregulated (Figure 1D, S2A,D). Surprisingly, these transcript-level changes in RNA expression generally were not reflected in the ribosome occupancy measurements. Lack of strong correlation between these data suggested that transcriptional changes had not propagated through translation. We were interested in measuring gene expression after a longer depletion to see if this discrepancy persisted. After 8 hours of IAA treatment, when eIF4E levels remain undetectably low (Figure 1C), we see greater agreement in the RNA abundance and ribosome occupancy measurements (Figure 1E, S3A–B). The improved correlation at 8 hours suggested that the disparities we saw after 1 hour of IAA treatment reflected pre-steady-state changes in translation. Although we saw larger changes in ribosome occupancy affecting more genes at the later timepoint, the total level of translation as measured by the HPG metabolic labeling assay recovered after 4 hours of eIF4E depletion despite unmeasurable levels of eIF4E (Figure 1B,C).

We hypothesized that the sensitivity of transcripts to eIF4E depletion may be influenced by certain RNA attributes, such as poly(A) length or 5’ UTR structure, based on known interactions between eIF4E, eIF4G, eIF4A, and Pab1 (Figure S2G, S3G). Correlations between published mRNA features and the changes we observed following eIF4E depletion yielded a relationship between mRNA stability and sensitivity to eIF4E depletion (Figure 1F, S2B-C, S3E) (Chan et al., 2018). Long-lived transcripts (those with a steady-state half-life greater than ~5 minutes) generally decreased in abundance following eIF4E depletion. The correlation between mRNA half-life and change in abundance following eIF4E depletion was also present after 1-hour IAA treatment (Figure S2C). Thus, these stability-related changes happened relatively quickly in response to eIF4E depletion, suggesting that eIF4E preferentially stabilizes those transcripts under unperturbed conditions. We also calculated correlations between RNA attributes and eIF4E-dependent changes in translation efficiency, which is measure calculated from ribosome footprints density changes given underlying RNA expression patterns. Changes in translation efficiency following eIF4E depletion most strongly correlated with transcript length (Figure 1G, S2E-F, S3F). Depletion of eIF4E reduces the translation efficiency of short mRNAs and depresses the length bias of translation efficiency. Overall, our findings suggested eIF4E promoted the expression of stable, short transcripts.

### Reduced translation drives aromatic amino acid accumulation and ARO10 induction

**Figure 2:**
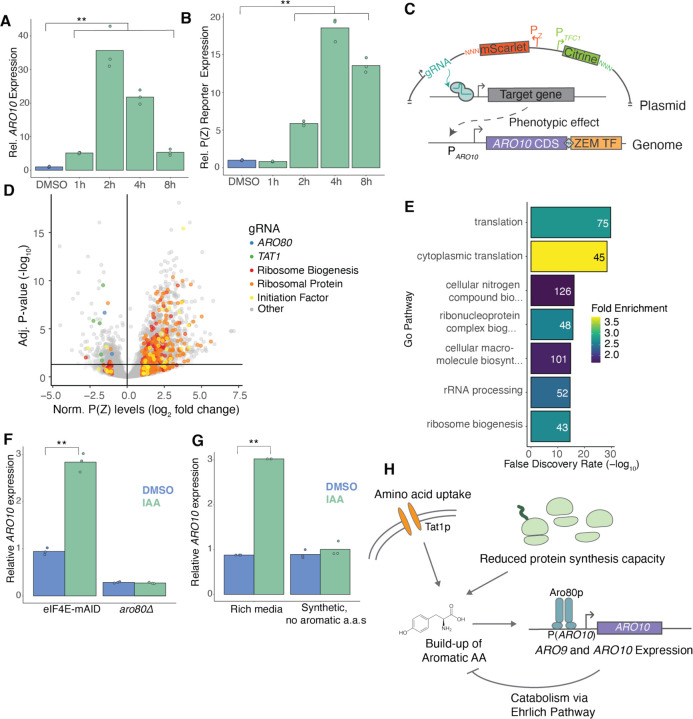
CiBER-seq profile *ARO10* expression regulation. **(A)** RT-qPCR of *ARO10* expression over the course of eIF4E-AID depletion. (**) represents p < 0.05 calculated by Student’s t test. **(B)** RT-qPCR of P(Z) reporter expression over the course of eIF4E-AID depletion. (**) represents p < 0.05 calculated by Student’s t test. **(C)** Schematic of CiBER-seq screen design. **(D)** Genome-wide CiBER-seq results showing fold-change (log_2_) in P(Z) reporter abundance, relative to P(*TFC1*) reporter levels, for each gRNA. Line indicates significance cutoff (adjusted p-value < 0.05). Color represents gene category or identity. **(E)** GO analysis for genes targeted by gRNAs that up-regulated P(Z) expression. gRNAs were filtered for fold-change (log_2_) > 1 and adjusted p-value < 0.05. The most statistically significant entries were chosen and narrowed based on percentage of overlapping genes. **(F)** RT-qPCR of *ARO10* expression following 1-hour of eIF4E-AID depletion in *ARO80* and *aro80Δ* cells. (**) represents p < 0.05 calculated by Student’s t test. **(G)** RT-qPCR of ARO10 expression following 1-hour of eIF4E-AID depletion in rich media and synthetic medium without aromatic amino acids. (**) represents p < 0.05 calculated by Student’s t test. **(H)** A model for *ARO10* upregulation and subsequent feedback in response to translational stress and aromatic amino acid availability in the medium.

While the above analysis revealed overall features associated with sensitivity to eIF4E depletion, the two genes most strongly upregulated following 1-hour eIF4E depletion, both at the level of RNA expression and ribosome occupancy, were *ARO9* and *ARO10*. These genes encode enzymes involved in the catabolism of aromatic amino acids via the Ehrlich pathway, which facilitates the utilization of aromatic amino acids as nitrogen sources under nitrogen-limiting conditions (Figure 1D) (Kradolfer *et al*. 1982; Vuralhan *et al*. 2003; Hazelwood *et al*. 2008). To better understand the gene regulatory network controlling *ARO9* and *ARO10* expression and its connection to cap-dependent translation factors, we established a reporter system to measure changes in *ARO10* transcription and translation. In this experimental setup, we fused a synthetic transcription factor (ZEM) to the endogenous *ARO10* CDS, separated by a self-cleaving P2A peptide (Aranda-Díaz et al., 2017). We contemporaneously introduced an RFP reporter under the control of an orthogonal promoter, p(Z), whose transcriptional output is proportional to ZEM protein abundance. Thus, we quantified *ARO10* transcription via *ARO10-ZEM* mRNA abundance, and indirectly reported on translation of *ARO10* by monitoring expression of RFP from the p(Z) promoter driven by the ZEM protein (Figure S4A). Our indirect reporter was induced to a similar degree by eIF4E depletion as endogenous *ARO10*, although its response was delayed slightly because reporter induction requires synthesis and nuclear import of the synthetic transcription factor (Figure 2A,B). Having established that this system recapitulated *ARO10* induction following eIF4E depletion, we set out to identify the regulatory network underlying this response.

To uncover the genetic regulatory network driving *ARO10* expression and the connections to eIF4E depletion, we used a genome-wide CRISPRi approach, based on CRISPRi with barcoded expression readout (CiBER-seq), that couples expression of our *ARO10* reporter protein via a unique RNA barcode to a specific genetic perturbation (Muller et al., 2020) (Figure 2C). We generated a library of approximately 48,000 gRNAs, each of which was linked to expressed RNA barcodes that could be tracked by next-generation sequencing. We measured barcode abundance from transcripts driven by the P(Z) reporter normalized against a paired barcode expressed from the housekeeping *TFC1* promoter, which allowed us to correct for knockdown effects that affect overall RNA transcription or cellular fitness. We sequenced the RNA barcodes before and after gRNA induction to understand how knockdown of factors changed *ARO10* reporter expression (Ashuach et al., 2019). As expected, guides targeting *ARO10* or *GAL1* (from which P(Z) is derived) were some of the strongest repressors of *ARO10* reporter expression, and we further validated the screen results by individually testing both a gRNA that reduced P(Z)-reporter when induced (*PAB1*) and a gRNA against *TIF34* which increases *ARO10* expression when induced (Figure S4E,F).

Our genome-wide screen revealed numerous gRNAs targeting ribosomal protein genes (RPGs) or genes involved in ribosome biogenesis (RiBi) that activated our *ARO10* reporter (Figure 2D). This pattern was reflected in GO analysis for gRNAs that significantly increased reporter expression (Figure 2E). This suggested to us that *ARO10* induction was not a unique response feature of eIF4E depletion, but instead was upregulated by any reduction of general translation machinery. Conversely, we found gRNAs targeting *ARO80*, a transcription factor known to regulate *ARO10*, that reduced *ARO10* expression (Figure 2D) (Lee and Hahn, 2013). Thus, we measured the *ARO10* response to eIF4E depletion in a strain lacking *ARO80* and found that, indeed, a*ro80*Δ abrogated *ARO10* induction (Figure 2F).

We also found several gRNAs that significantly reduced *ARO10* expression by targeting components of the SPS-sensing pathway, which regulates the transcription of amino acid permeases in response to extracellular amino acids (Ljungdahl, 2009) (Figure S4H,I). While *ARO10* could be a direct, but unreported, transcriptional target of the SPS pathway, we considered whether this could instead reflect an indirect effect. To explore this possibility, we searched for gRNAs against annotated targets of SPS transcription factors Stp1 or Stp2 that significantly reduced *ARO10* reporter expression (Eckert-Boulet et al., 2004). Knockdown of *TAT1*, a known target of Stp1 and Stp2 that encodes an amino acid transporter for tyrosine, leucine, isoleucine and valine, reduced *ARO10* expression (Bajmoczi et al., 1998; Schmidt et al., 1994). Notably, Tat1 imports the major substrates for Aro9 and Aro10, and Aro80 is allosterically activated by tryptophan (Lee and Hahn, 2013). Thus, SPS control of aromatic amino acid import may explain these effects on *ARO10* expression. To test the possibility that the import of extracellular amino acids mediated *ARO10* expression, we grew cells in synthetic media lacking aromatic amino acids and found that this condition indeed mitigated the induction of *ARO10* after eIF4E depletion (Figure 2G). Taken together, these findings suggest that the buildup of aromatic amino acids imported by Tat1 but not needed for protein synthesis is sensed by Aro80, which upregulates *ARO9* and *ARO10* to catabolize them (Figure 2H).

We reasoned that this catabolism might mitigate toxicity induced by excess aromatic amino acids or produce a metabolite needed for adaptive responses to reductions of protein synthesis. However, neither deletion of *ARO9* nor of *ARO80* caused any difference in growth rate upon eIF4E depletion. (Figure S5A,B). Thus, the induction of the Ehrlich pathway enzymes is not required in cells depleted of eIF4E.

Surprisingly, long-term eIF4E depletion led to the up-regulation of *ARO1* (Figure S3B), which encodes a multi-functional chorismite biosynthesis enzyme required to produce aromatic amino acids (Duncan et al., 1988). Induction of this biosynthetic enzyme, along with continued expression of the opposing catabolic pathway, suggested that eIF4E depletion led to futile or gratuitous metabolism. Indeed, we found that cells depended on aromatic amino acid biosynthesis after eIF4E depletion, as shown by slowed growth of *aro1Δ* cells (Figure S5C). Furthermore, the negative synthetic interaction between eIF4E depletion and *aro1Δ* was partially rescued by supplementing media with additional aromatic amino acids (Figure S5D). Combined, these results show that eIF4E depletion disrupts amino acid homeostasis.

### eIF4E depletion activates *GCN4* translation in *GCN2*-independent manner

**Figure 3:**
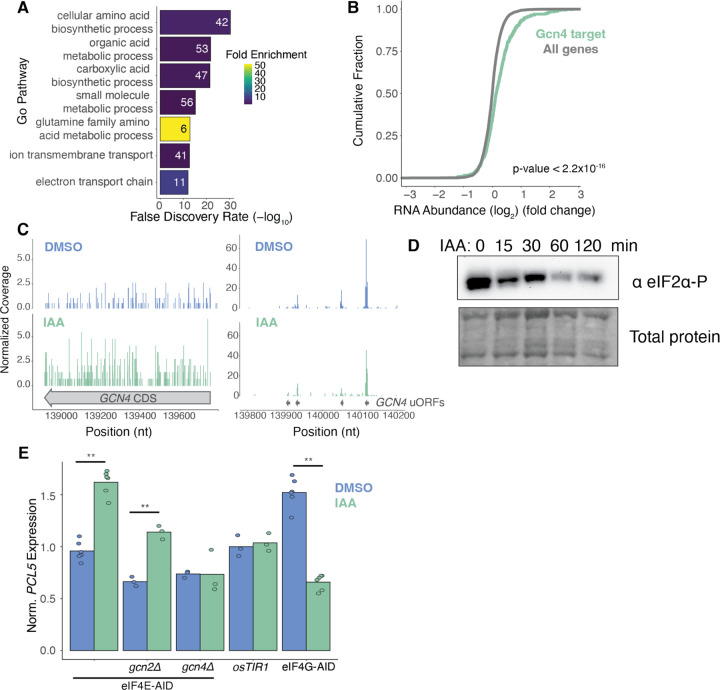
*GCN4* activation in response to eIF4E depletion. **(A)** GO analysis for genes which were significantly (adj. p-value < 0.05) up-regulated following eIF4E-depletion (8-hour treatment) in RNA-seq analysis. The most statistically significant entries were chosen and narrowed based on percentage of overlapping genes. **(B)** Empirical cumulative distribution function showing relationship between change in mRNA expression following eIF4E depletion for genes categorized as Gcn4 transcriptional targets. P-value was calculated using the Mann-Whitney U test. **(C)** Ribosome footprints (adjusted to A-site) over the GCN4 locus for 8-hour IAA and DMSO control cells. Read counts were scaled based on library size. **(D)** Western blot for eIF2α phosphorylation levels over the course of IAA depletion. **(E)** RT-qPCR of *PCL5* expression following 1-hour of IAA treatment. (**) represents p < 0.05 calculated by Student’s t test.

Indeed, we saw broad up-regulation of amino acid biosynthesis pathways following 8 hours of eIF4E depletion, indicating a breakdown in coordination between amino acid levels and the regulation of genes involved in amino acid biosynthesis (Figure 3A). These biosynthetic genes comprise the regulon of Gcn4, a transcription factor that is itself translationally induced in response to amino acid starvation (Hinnebusch, 2005). Indeed, RNA abundance of Gcn4 target genes increased after 8 hours of IAA treatment compared to non-target genes (Figure 3B). We also found a modest but significant translational upregulation of Gcn4 (log_2_ fold-change = 0.61, adj. *p*-value = 1.1×10^−4^) after 1 hour of eIF4E depletion, as measured by ribosome occupancy (Figure 3C).

The translational induction of *GCN4* in response to amino acid starvation has been well characterized (Hinnebusch, 2005). During non-starvation conditions, the expression of *GCN4* is suppressed via repressive uORFs in the 5′UTR. This repression is relieved when the cell experiences amino acid starvation. When protein synthesis outstrips amino acid availability, uncharged tRNAs accumulate, causing the kinase Gcn2 to phosphorylate the translation initiation factor eIF2α. Phosphorylation of eIF2α reduces the availability of the active form of eIF2 bound to initiator tRNA, which allows reinitiating ribosomes in the *GCN4* 5’ UTR to bypass uORFs and reach the *GCN4* CDS, increasing Gcn4 protein levels (Dever et al., 1992). Surprisingly, although *GCN4* activation is canonically accompanied by an increase in eIF2a phosphorylation, we found that levels of eIF2α phosphorylation decreased over the course of eIF4E depletion (Figure 3D). Phosphorylation may decrease because reduced translation during eIF4E depletion reduced ribosomal collisions, another trigger for Gcn2 activation (Wu et al., 2020); in any case, the increased *GCN4* translation that we observed could not be explained by the Gcn2-eIF2α pathway.

To further dissect the genetic requirements for the novel mechanism of *GCN4* activation in eIF4E-depleted cells, we monitored the expression of the Gcn4 target gene Pho85 cyclin 5 (*PCL5)* in *gcn2Δ* and *gcn4Δ* cells (Shemer et al., 2002) (Figure 3E). We found that *PCL5* induction was Gcn4-dependent but Gcn2-independent, consistent with the lack of eIF2α phosphorylation (Figure 3E). To test whether translation deficiencies in general activated Gcn4 and thereby increased *PCL5* expression, we integrated AID tags at both paralogs of the translation initiation factor eIF4G, allowing us to conditionally deplete this essential translation factor. Depleting eIF4G significantly decreased *PCL5* expression, in contrast to the effects of eIF4E depletion (Figure 3E). We therefore concluded that the non-canonical Gcn4 activation following eIF4E depletion was not a result of general deficiencies in translation, but instead was a specific response to eIF4E-depletion.

### PCL5 *translation is regulated by uORFs and poly(A) tract in 5′ UTR*

**Figure 4:**
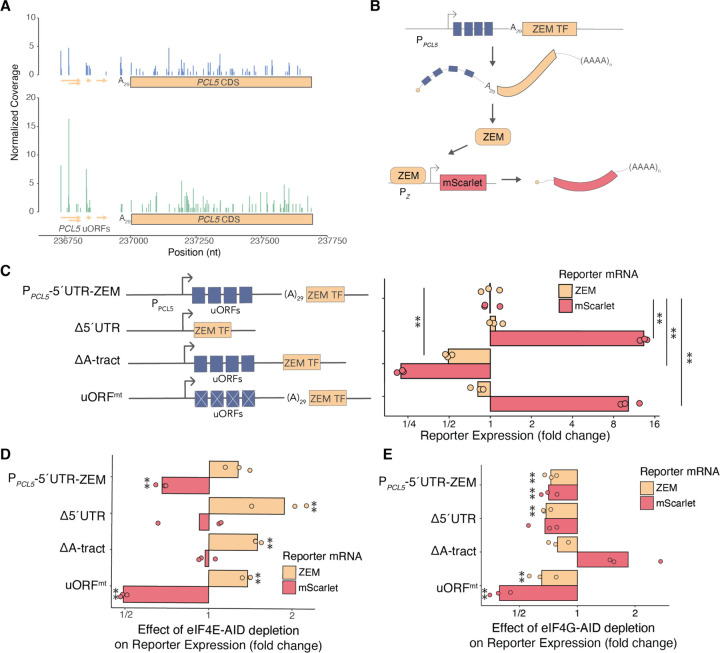
Translation regulation of *PCL5*. **(A)** Ribosome footprints (adjusted to A-site) over the *PCL5* locus for 8-hour IAA and DMSO control cells. Footprint counts were scaled based on library size and not adjusted for PCL5 abundance. **(B)** Schematic of *PCL5*-ZEM reporter assay. **(C)** RT-qPCR of ZEM transcript and mScarlet reporter expression of *PCL5* 5′UTR mutants normalized to 5′UTR^WT^ reporter. (**) represents p < 0.05 calculated by Student’s t test. **(D)** RT-qPCR of ZEM transcript and mScarlet reporter expression of *PCL5* 5′UTR mutants following eIF4E-AID depletion. Individual reporters were normalized to un-depleted control. (**) represents p < 0.05 calculated by Student’s t test. **(E)** Same as (D) except in eIF4G-AID depletion.

*PCL5* is a notable Gcn4 transcriptional target because it mediates a negative feedback circuit regulating Gcn4 activity (Shemer et al., 2002). The Pcl5 cyclin activates the cyclin-dependent kinase Pho85 to phosphorylate Gcn4, thereby marking it for degradation (Shemer et al., 2002). We reasoned that Gcn4 upregulation in eIF4E-depleted cells may reflect a breakdown in this negative-feedback loop. Curiously, when we evaluated the sequence of the 5′UTR of *PCL5*, we noted the presence of two potential uORFs with AUG start codons, followed by a startling stretch of 29 consecutive A bases—the longest such tract in the *S. cerevisiae* transcriptome (Vopálenský et al., 2019). Previous studies had also noted these potential AUG-start sites based on their conservation in yeast and the *PCL5* transcript structure, but they have heretofore been uncharacterized as regulators of *PCL5* translation (Cvijović et al., 2007; Zhang, 2005). The uORFs in the *PCL5* 5′UTR were reminiscent of the regulatory uORFs in the *GCN4* 5′UTR, which are responsible for its translational repression in un-starved conditions. The presence of *PCL5* uORFs could have important implications for Pcl5 protein synthesis during amino acid deprivation and therefore in the control of Gcn4 activity. By mapping ribosome occupancy across the 5′UTR of *PCL5*, we confirmed the translation of the two predicted AUG uORFs and, surprisingly, also observed translation of two uORFs beginning with non-cognate UUG codons (Figure 4B).

Interestingly, although *PCL5* was transcriptionally upregulated in response to eIF4E depletion (Figure 3E), we did not find a corresponding increase in ribosome occupancy over the *PCL5* ORF (Figure 4A). Instead, ribosome density in the *PCL5* 5′UTR was increased at all four uORFs in eIF4E-depleted cells, but most strikingly at the UUG uORF start codons (Figure 4A). In full, the translation efficiency of *PCL5* was reduced over 3-fold in response to eIF4E depletion (log_2_ fold-change = −1.77). We hypothesized that the distinctive regulatory features in the *PCL5* 5′UTR may have reduced its translation in eIF4E-depleted cells and sought to further characterize this regulation given the role of Pcl5 in repressing Gcn4 activity.

To further investigate how these unique *cis* features regulate *PCL5* translation, we designed fluorescent protein reporters fused to mutant versions of the *PCL5* 5′UTR, each testing the contribution of different 5′ UTR elements (Figure S6A). Unfortunately, most of reporters were weakly expressed and could not be distinguished from background fluorescence by flow cytometry (Figure S6A). We thus turned again to an indirect reporter system using the ZEM synthetic transcription factor, which amplifies signal from weakly expressed reporters (Aranda-Díaz et al., 2017) (Figure 4B). In this system, we measured changes to *PCL5-*reporter transcription and mRNA stability by tracking ZEM transcript levels (Figure 4B). Additionally, we measured *PCL5*-reporter translation indirectly, by measuring the abundance of a ZEM-driven transcriptional mScarlet reporter (Figure 4B). We generated four versions of this *PCL5-*ZEM reporter, each with the native *PCL5* promoter. We compared the full-length, wild type *PCL5* 5′UTR with three variants removing some or all of its distinctive *cis-*elements (Figure 4C, S6B). We then measured ZEM and mScarlet mRNA abundances to report on *PCL5* transcription and translation efficiencies, respectively. The *PCL5* uORFs suppressed translation of the ZEM transcription factor, consistent with the repressive effects typically seen from uORFs (Figure 4C, S6B). Furthermore, deletion of the poly-(A) tract reduced ZEM transcript levels by approximately 50%, and mScarlet transcript levels by 75%, relative to wild type (Figure 4C, S6B), suggesting that the long polyA tract in the *PCL5* 5′UTR enhanced its stability and translation.

Our previous findings indicated differential regulation of *PCL5* transcript levels in response to defects in translation initiation; Gcn4-dependent upregulation following eIF4E depletion and downregulation following eIF4G depletion (Figure 3E). To investigate whether *PCL5* translation was similarly affected by defects in translation initiation, we tracked changes to mRNA levels in these reporter constructs following depletion of eIF4E and eIF4G (Figure 4D,E, S6C-F). We observed an increase in ZEM-reporter transcript levels in all *PCL5-*ZEM reporters following eIF4E depletion, regardless of 5′UTR composition, likely mediated through the previously described *GCN4* response (Figure 4D, S6C,D). However, we found a decrease in mScarlet translation following eIF4E depletion in both the full-length 5′UTR reporter construct and the uORF mutation reporter.

In agreement with our measurements of endogenous *PCL5* transcript levels, eIF4G depletion consistently reduced ZEM-reporter transcript levels in all *PCL5-*ZEM reporters (Figure 4E, S6E,F). Strikingly, ZEM mRNA abundance was reduced even in the reporter that lacked the *PCL5* 5′UTR (Figure 4E, second line), suggesting that the regulation of *PCL5* in response to eIF4G depletion was transcriptional. Furthermore, we noted that expression of the reporter containing all four uORFs but lacking the polyA tract— the most repressive 5′UTR under wild-type conditions—was markedly less repressive following eIF4G-depletion. We therefore concluded that eIF4G may have played a role in suppressing translation in that context.

### Dissecting the genetic requirements for regulation of *PCL5* expression

**Figure 5:**
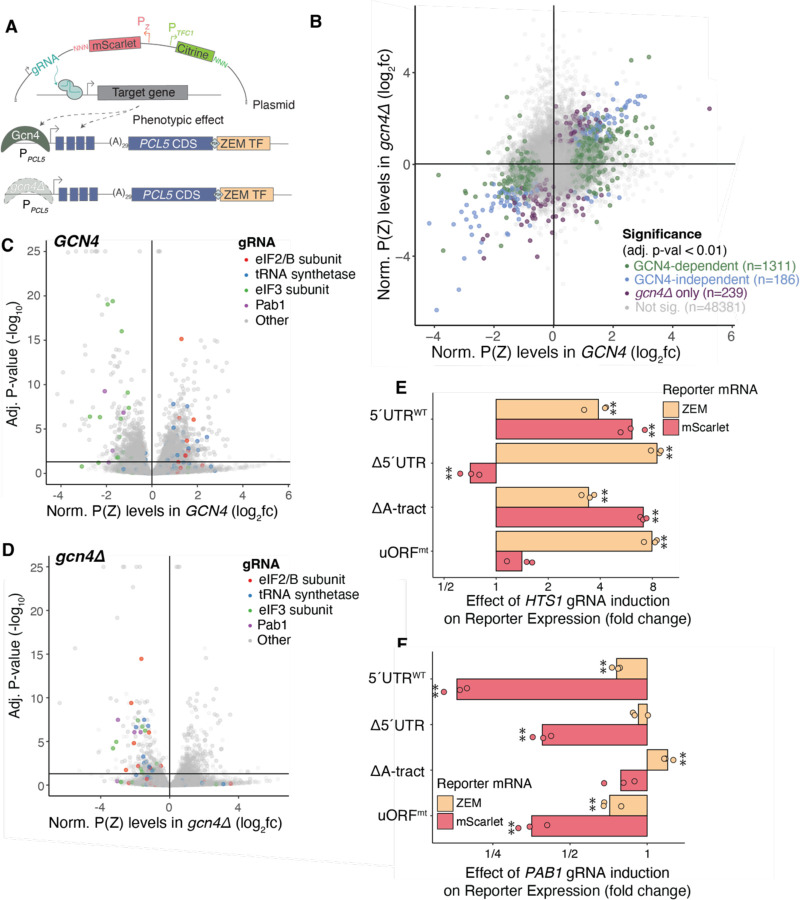
CiBER-seq genetic screen for regulators of *PCL5* expression. **(A)** Schematic of CiBER-seq screen design. **(B)** Genome-wide CiBER-seq screen results showing fold-change (log_2_) in P(Z) reporter abundance, relative to P(*TFC1*) reporter levels for each gRNA in *GCN4* and *gcn4Δ* backgrounds. Color indicates significance cutoff (adjusted p-value < 0.05). **(C)** Genome-wide CiBER-seq screen results showing fold-change (log_2_) in P(Z) reporter abundance, relative to P(*TFC1*) reporter levels for each gRNA in *GCN4* background. Line indicates significance cutoff (adjusted pvalue < 0.05). Color represents gene category or identity. **(D)** Same as (C) in *gcn4Δ*. **(E)** RT-qPCR of ZEM transcript and mScarlet reporter expression of *PCL5* 5′UTR mutants following HTS1 gRNA induction. Individual reporters were normalized to uninduced control. (**) represents p < 0.05 calculated by Student’s t test. **(F)** Same as (E) except following *PAB1* gRNA induction.

Although the 5′UTR of *PCL5* contained sequence features that modulated its translation, we found that depleting eIF4E and eIF4G primarily affected its transcription rather than its translation. Thus, we investigated the potentially novel mechanisms regulating the translation of this factor by CiBER-seq profiling, using an indirect reporter of the same design as our *ARO10* reporter (Figure 2) (Muller et al., 2020). By fusing the ZEM transcription factor to the endogenous Pcl5 with a P2A self-cleaving peptide between the two proteins, we were able to capture regulators of *PCL5* transcription and translation using a transcriptional readout (Figure 5A). To parse the Gcn4-dependent transcriptional effects on *PCL5* from the translation regulation imparted through its 5′UTR, we performed parallel CiBER-seq analyses in a wild-type *GCN4* genetic background and one in which *GCN4* was deleted (*gcn4Δ)* (Figure 5A).

Our screen identified thousands of guides that affected *PCL5* expression. Notably, the majority of these guides (1311/1780) had Gcn4-dependent effects (Figure 5B) and overlapped with previously published CiBER-seq analysis of *GCN4* itself (Figure S7H,I) (Muller et al., 2020). Many gRNAs known to activate the integrated stress response (ISR), such as those targeting tRNA synthetases and eIF2 or eIF2B subunits, induced *PCL5* expression in the *GCN4* wild type cells (Figure 5C). Interestingly, in *gcn4Δ* cells, these same gRNAs repressed *PCL5* expression (Figure 5D). This finding was surprising as we had anticipated similar regulation of *PCL5* and *GCN4* given the similarities between their 5′ UTRs. To further validate this finding, we tested a gRNA against the histidinyl-tRNA synthetase *HTS1*, whose knockdown is known to activate the ISR, in our *PCL5-ZEM* reporter system (Figure 5E, S8B,E). Consistent with results from our screen, knockdown of *HTS1* increased ZEM transcript levels and protein output (Figure 5E, S8B,E). Furthermore, we saw uORF-dependent regulation that maintained *PCL5* translation during ISR activation; reporters lacking the uORFs showed transcript-level increases that were not matched by increased protein output. (Figure 5E, S8B,E). This finding demonstrates for the first time that the translation of *PCL5* is regulated much like the translation of *GCN4* translation in response to eIF2a phosphorylation (Dever et al., 1992). These findings appeared to contradict our results from the *gcn4Δ* screen for regulators of *PCL5* translation (Figure 5A,D). To reconcile the two results, we hypothesize that there is a mechanism for suppressing *PCL5* expression when cells are unable to mount a sufficient Gcn4 response to ISR activation. Our screen recapitulated the *GCN4-*dependent induction of *PCL5* in response to loss of eIF4E and a contrasting, *GCN4-*independent reduction when eIF4G was depleted. We also observed system-dependent effects from knockdown of *PCL5* itself, the *GAL1*-derived reporter, and the *TFC1* normalizer (Figure S7F,G).

We were particularly interested in hits from our screen that regulated *PCL5* expression independently of Gcn4, as these may reveal mechanistic insight into translation control of *PCL5*. Knockdown of *PAB1*, which encodes the yeast poly-(A) binding protein, caused a *GCN4*-independent decrease in Pcl5 (Figure 5D)—a striking observation in light of the 29 base poly-(A) tract in the *PCL5* 5′ UTR, which is long enough to bind Pab1 (Sachs et al., 1987). Indeed, we tested the effects of *PAB1* knockdown on mutant *PCL5* reporters and found that removal of the poly-(A) tract greatly attenuated the strong translational repression seen in the wild-type version (Figure 5F, S8D,G). We likewise observed that guides targeting subunits of the translation initiation factor eIF3 reduced *PCL5* reporter translation even in the absense of *GCN4*. As eIF3 has known roles in ribosome recycling and re-initiation, particularly in the context of the uORFs in the *GCN4* 5′UTR (Sonenberg and Hinnebusch, 2009), we confirmed that knockdown of eIF3 subunit gene *NIP1* reduced reporter mRNA and translation levels in a uORF-dependent manner (Figure S8A,C,F). These results, which matched our CiBER-seq data, suggested that eIF3 supports translation reinitiation downstream of *PCL5* uORFs as well. Together, our findings highlighted the complexity of mechanisms governing *PCL5* expression and suggested that regulation of *PCL5* serves as an integration point of different signaling pathways to sculpt the Gcn4 response.

## Discussion

We showed that acute depletion of the essential cap-binding protein eIF4E had surprisingly modest effects on cell growth and protein synthesis. Although eIF4E levels were quickly reduced below 10% of starting abundance, we observed a relatively uniform 50% reduction in overall translation. We did note more nuanced changes to mRNA abundance, including a strong reduction in the expression of long-lived transcripts, likely reflecting more rapid turnover (Chan et al., 2018). There are conflicting data on whether the eIF4E-mRNA interaction is rate-limiting for cap-dependent translation initiation (Firczuk et al., 2013; von der Haar et al., 2004). Furthermore, eIF4E concentration exceeds that of mRNA several-fold, while eIF4G has roughly matched stoichiometry with mRNA. Notably, this implies the majority of eIF4E is not in complex with eIF4G and may be stabilizing or promoting translation of specific transcripts (Firczuk et al., 2013; Krause et al., 2022). In contrast to our results, prior work reported that transcriptional titration of eIF4E led to proportional decreases in protein synthesis and growth rates (Firczuk et al., 2013; von der Haar et al., 2004). In mice, however, eIF4E haploinsufficiency does not cause bulk translation defects (Truitt et al., 2015) , suggesting that the relative insensitivity to loss of cap-binding protein might be a conserved aspect of translation biology (O’Leary et al., 2013; von der Haar and McCarthy, 2002).

We further showed that protein synthesis recovered after only four hours, although eIF4E levels remained extremely low, suggesting that cells robustly adapted to this condition. Following prolonged eIF4E depletion, cells exhibited more substantial changes to translation than seen at earlier timepoints. These long-term translation changes weakened the bias towards more efficient translation of short transcripts. This trend is intriguing given that efficient translation of short transcripts is attributed to the formation of a closed-loop structure, wherein eIF4G bridges between eIF4E at the 5′ end of an mRNA and the poly(A)-binding protein, Pab1, at its 3′ end (Amrani et al., 2008; Çetin and O’Leary, 2022; Costello et al., 2015; Thompson and Gilbert, 2017). Depletion of eIF4E would abolish closed-loop formation, explaining the reduced the translation efficiency of short mRNAs and depressed the length bias of translation efficiency.

The strongest gene-specific effects of eIF4E depletion arose as secondary effects of reduced protein biosynthesis on amino acid pools. The transcription factor Aro80 responded to changes in translational status by activating a strong catabolic response through Ehrlich pathway enzymes Aro9 and Aro10, a gene expression program observed under different starvation stress conditions (Staschke et al., 2010; Xia et al., 2022; Zou et al., 2020). In the context of eIF4E depletion, this catabolic response appears maladaptive as cells require ongoing aromatic amino acid biosynthesis or supplementation to grow. It may play other roles, however—aromatic alcohols produced by this pathway serve as quorum sensing molecule in *S. cerevisiae* (Chen and Fink, 2006; Zhang et al., 2021).

Paradoxically, reductions in eIF4E activity also caused translational activation of *GCN4* and thereby induced a transcriptional response that favored amino acid anabolic processes. Surprisingly, this activation of *GCN4* occurred independently of Gcn2 and without eIF2α phosphorylation, indicating the involvement of a non-canonical mechanism. Fusel alcohols produced by amino acid catabolism can regulate *GCN4* translation through the inhibition of eIF2B, acting downstream of eIF2α phosphorylation, offering one possible explanation (Ashe, 2001; Hazelwood et al., 2008). Beyond this possibility, we have recently described a range of genetic perturbations to translation that lead to Gcn2-independent *GCN4* induction (Muller et al., 2020). In the case of eIF4E depletion, reduced recruitment of ribosomal 40S subunits for translation could increase the availability of free 40S subunits and thereby depress the ratio of eIF2/40S, similar to the *GCN4* activation caused by large subunit biogenesis (Steffen et al., 2008).

The translational repression of *PCL5*, a negative regulator of Gcn4, may have further imbalanced amino acid pools following eIF4E depletion. Pcl5 promotes Gcn4 degradation. Because *PCL5* expression is induced by Gcn4, this regulated degradation serves as a feedback mechanism that controls Gcn4 activity (Shemer et al., 2002). Feedback control requires that Pcl5 protein synthesis is proportional with its Gcn4-driven transcription, but maintaining the correspondence between mRNA levels and protein production poses a challenge when translation is impaired due to intrinsic limitations, inhibition by eIF2α phosphorylation, or other factors—exactly the conditions when Gcn4 regulation is needed. The requirement for uniform translation under varying conditions may explain why *PCL5* contains uORFs similar to those that control *GCN4* translation. Our findings highlight the significance of these characteristics as a point of regulation for *PCL5* that shapes the integrated stress response in yeast.

The post-transcriptional control of *PCL5* has distinctive characteristics, however, which may be mediated through the poly(A) tract in its 5′ UTR. Our finding that the poly(A) tract and Pab1 stabilize the *PCL5* transcript is reminiscent of previous work that found internal Pab1-tethering recruited the termination factor eRF3 and stabilized nonsense-containing mRNAs (Amrani et al., 2004). Additionally, *PCL5* translation regulation may integrate additional signals about cellular environment, such as heat shock, a condition in which Pab1 has been shown to phase separate (Riback et al., 2017). While poly(A) tracts have previously been linked to cap-independent translation of internal ribosome entry sites in yeast (Gilbert et al., 2007), translation of *PCL5* appears to be cap-dependent; it is repressed by uORFs, which affect cap-dependent scanning, and it is reduced upon depletion of cap-binding eIF4E (Figure 4).

**Figure 6:**
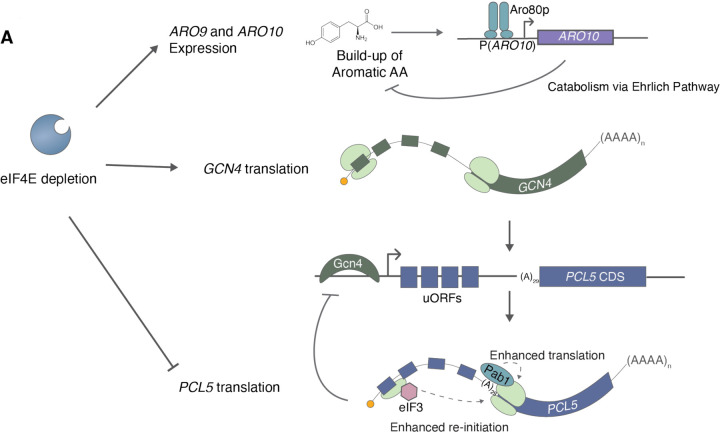
Model of the role of eIF4E in maintenance of amino acid homeostasis. **(A)** Model emphasizing the effects of eIF4E depletion on dysregulation of amino acid metabolism gene expression and mechanisms of *PCL5* translational regulation.

Amino acid homeostasis is crucial for cell growth and survival. The tight regulation of amino acid pools involves a complex interplay between amino acid uptake, biosynthesis, incorporation into protein, and catabolism. Hundreds of genes are regulated response to changes in amino acid availability. Our study has identified the cap-binding protein eIF4E as a central coordinator of both biosynthetic and catabolic gene expression (Figure 6). Depletion of eIF4E led to upregulation of both opposing metabolic processes, resulting in an excess of aromatic amino acids and a discordant upregulation of amino acid biosynthesis genes, potentially indicative of gratuitous or futile amino acid metabolism. Intriguingly, mammalian cells couple amino acid sensing with protein synthesis through cap-centric regulation as well, albeit through a distinct mechanism. Indeed, dysregulation of eIF4E and metabolism are hallmarks of cancer, emphasizing the importance of understanding the underlying mechanisms to develop effective therapeutic strategies.

## Materials and Methods

### Yeast strains

Strains used in this study are listed in Table S1. All strains were derived from the S. cerevisiae BY4742 using standard genetic techniques and CRISPR-Cas9 technology (Lee et al., 2015). C-terminal mAID-3xFlag tags (cdc33-mAID-3xFlag, tif4631-mAID-3xFlag) were generated by amplifying the SG_linker-mAID-3xFlag sequence from pNTI433 with primers that included 40 bp of sequence identity with either side of the insertion, then integrated using CRISPR-Cas9 technology as described by (Brothers and Rine, 2019). C-terminal mAID-3xV5 tag (tif4632-mAID-3xV5), and P2A-ZEM tags (aro10-P2A-ZEM and pcl5-P2A-ZEM) were constructed in the same manner by amplifying from synthetic DNA gene block (Integrated DNA Technologies) PD552 and pNTI730, respectively. Gene deletions were generated using one-step replacement with marker cassettes, more specifically by PCR amplification of the hygromycin resistance cassette from pNTI730 with flanking sequence homology to the 5’ and 3’ ends of target gene coding sequence (Goldstein and McCusker, 1999; Hentges et al., 2005). *PCL5*-ZEM reporters and dCas9-TetR were integrated using NotI-linearized vectors from the EasyClone 2.0 toolkit for yeast genomic integration (Stovicek et al., 2015). Plasmids and primers used in strain construction are listed in Table S2 and S3, respectively.

### Plasmids

Plasmids used in this study are listed in Table S2. For CRISPR-Cas9 editing, guide RNA target and nontarget strands were integrated into single guide RNA dropout-Cas9 expression plasmid (pJR3429, a gift from the Rine lab) as described in (Brothers and Rine, 2019). Annealing oligos for gRNA insertion are listed in Table S3. The PCL5-ZEM reporter plamids (PDp86–89) were made using standard Gibson cloning into pCfB2337 (Gibson et al., 2009; Stovicek et al., 2015).The PCL5-mCherry reporter plasmids (PDp74–78) were made using standard Gibson cloning into pRS315 LEU2 CEN/ARS (a gift from the Rine lab). P(*Z*)-mScarlet and P(*TFC1*)-citrine reporter plasmids with individual guide RNAs were made through standard Gibson cloning of annealed oligos into a single isolate of the plasmid library.

### HPG assay

Yeast cells were inoculated in a custom turbidostat (McGeachy et al., 2019) and maintained in SCD -Met media at a density corresponding to OD_600_ of 0.6. After growth rate reached a steady state, indole-3-acetic acid (IAA) (Sigma-Aldrich #12886) was injected into the growth chamber and media reservoir to achieve a final concentration of 500 *μ*M. After indicated duration of eIF4E depletion, 5 mL of cells were collected for western blot analysis and HPG nascent peptide labeling.

For HPG nascent peptide labeling assay, cells were back-diluted to OD_600_ of 0.3 in SCD -Met with 500 *μ*M IAA and 50 *μ*M L-Homopropargylglycine (HPG) (Thermo Fisher Scientific #C10186). Nascent peptide labeling was conducted for 2 hours at 30°C. For cycloheximide control samples, 50 *μ*g/mL cycloheximide (Sigma-Aldrich #C4859) was added concurrently with HPG. Cells were harvested by centrifugation and fixed at room temperature while nutating in 4% paraformaldehyde (Electron Microscopy Sciences #15710). Cells were washed with PBS with 2mM EDTA and permeabilized at 45°C with 2% w/v sodium lauroyl sarcosine (Bioworld #41930024–3) in PBS for 15 minutes (Abraham and Bhat, 2008). Click-iT^™^ chemistry was performed per manufacturer’s recommendations (Thermo Fisher Scientific #C10428) to label HPG with Alexa Fluor^™^ 488 azide. Fluorescence was measured by flow cytometry analysis on a BD LSR Fortessa X20 with excitation by the 488 nm blue laser, captured on the FITC channel.

### Polysome profiling

Cells were grown to mid-log phase (typically 100 mL cultures at OD_600_ 0.6 – 1.0) in YPD, harvested by filtration with 0.45 *μ*M Whatman cellulose nitrate membrane filters (GE Life Sciences #7184–004). 100 *μ*g/mL cycloheximide was added to the culture immediately before filtration. Samples were lysed by cryogrinding with the MM400 Mixer mill (Retsch #20.745.0001) in polysome buffer (20 mM Tris-HCl pH 7.4, 150 mM NaCl, 5 mM MgCl_2_, 1 mM DTT, 100 *μ*g/mL cycloheximide), followed by centrifugation at 10,000 g for 10 min at 4°C. The supernatant was collected and stored at −80°C.

Sucrose gradients were prepared using a Gradient Master (BioComp Instruments, Fredericton, Canada). Normalized lysates (200 *μ*L) were loaded onto a 10–50% sucrose gradient prepared in polysome buffer. Gradients were centrifuged at 36,000 rpm for 4 hours at 4°C in a Beckman SW41Ti rotor. The gradient run through the Gradient Master, and the absorbance at 254 nm was monitored continuously using a BioRad EM-1 Econo UV Monitor.

### Western blotting

Cells were grown to mid-log phase (typically 5 mL cultures at OD_600_ 0.6 – 1.0) in YPD, harvested by centrifugation, and washed with 20% TCA (Cox et al., 1997). The pellet was flash frozen in liquid nitrogen and stored at −80°C until further use. For lysis and extraction, the pellet was thawed on ice and resuspended in 20% TCA (Sigma-Aldrich #T6399) and glass beads (Sigma-Aldrich #Z250465) The sample was then lysed by vortexing and centrifuged at 4°C, 20,000xg for 10 minutes. The pellet was washed with ice-cold 100% ethanol and centrifuged again. The final pellet was resuspended in Tris pH 8 buffer.

Equal amounts of lysate were denatured for 10 minutes at 80°C and loaded on a 4 to 12% polyacrylamide Bis-Tris gel (Thermo Scientific #NW04120BOX). The gel was run at 120V for approximately 80 minutes in MOPS buffer. Protein was then transferred to a nitrocellulose membrane (Thermo Scientific #88018) according to manufacturer’s guidelines. Membranes were blocked for 1 hour in TBST with 0.5% milk. Primary antibodies were incubated with the membrane overnight at 4°C while shaking and secondary antibodies were incubated for 1 hour at room temperature. Membranes were washed three times by shaking for 5 minutes in TBST following primary and secondary incubations. All blots were developed with Pierce ECL Western Blotting Substrate (Thermo Scientific #32209) and imaged by a FluorChem R imaging system (ProteinSimple). The antibodies and corresponding dilution factors used in this study include rabbit anti-Flag (CST #2368S) (1:1000), rabbit anti-V5 (CST #13202) (1:1000), rabbit anti-HA (CST #3724) (1:1000), mouse anti-GAPDH (Proteintech #60004–1-IG) (1:5000), rabbit anti-Phospho-eIF2α(Ser51) (CST #3398S) (1:1000), HRP-conjugated anti-rabbit IgG (CST #7074) (1:5000).

### Ribosome profiling and RNA-sequencing

Cells were treated with 500 *μ*M indole-3-acetic acid (IAA) (Sigma-Aldrich #12886) or DMSO for either 1 or 8 hours and grown to mid-log phase (typically 150 mL cultures at OD_600_ 0.6 – 1.0) in YPD. Yeast cells were harvested by filtration with 0.45 *μ*M Whatman cellulose nitrate membrane filters (GE Life Sciences #7184–004). Ribosome profiling was conducted as detailed in (McGlincy and Ingolia, 2017) for 1-hour eIF4E depletion samples and corresponding DMSO controls. Adjustments were made to (McGlincy and Ingolia, 2017) for the 8-hour eIF4E depletion samples and their corresponding DMSO control samples as follows. Reverse transcription was performed with primer PD1131. rRNA depletion was achieved via subtractive hybridization was performed using complementary oligos PD1043–1046 as described in (Ingolia et al., 2012). Circularization was performed with CircLigase I (Epicentre #CL4111K) instead of CircLigase II, following the manufacturer’s protocol recommendations.

Total RNA for RNA-sequencing was isolated from cell lysates using acid phenol extraction (Ares, 2012). For 1-hour eIF4E depletion samples and corresponding DMSO controls, poly(A) enrichment was performed using Dynabeads^™^ oligo(dT)_25_ (Thermo Scientific #61002) according to the manufacturer instructions. For 8-hour eIF4E depletion samples and corresponding DMSO controls, rRNA depletion was performed using QIAseq FastSelect -rRNA yeast kit (Qiagen #334215) according to the manufacturer instructions. All libraries were generated with NEBNext Ultra II Directional RNA library prep kit (NEB #E7760).

Reads from ribosome profiling were processed as detailed in (McGlincy and Ingolia, 2017). Ribosome profiling and RNA-seq reads were aligned using STAR (Dobin et al., 2013) to the S288C reference genome R64.1. Differential expression analysis was performed with DESeq2 (Love et al., 2014).

### Plasmid library generation and transformation

The divergent *P(Z)-*mScarlet and *P(TFC1)-*citrine plasmid libraries were generated as detailed in (Muller et al., 2022, 2020) with the following modifications. The divergent promoter inserts for Gibson assembly (NEB #E2621L) were generated by digesting PDp82 with SalI and AvrII. These libraries were transformed into ElectroMax DH10B cells (Thermo Scientific, #18290015) by electroporation according to manufacturer’s protocol. Serial dilutions of transformations were plated to ensure sufficient library diversity (>50x coverage of 240,000 unique barcodes).

Plasmid libraries were transformed into yeast using standard lithium acetate transformation (Gietz and Schiestl, 2007). Serial dilutions of transformations were plated to ensure sufficient library diversity (>5x coverage of 240,000 unique barcodes).

### CiBER-seq

Yeast populations transformed with plasmid libraries were inoculated in a custom turbidostat (McGeachy et al., 2019) and maintained in SCD -URA media with 10 nM beta-estradiol at a density corresponding to OD_600_ of 0.6. After a period of six doublings (9 hours), a sample of 50 mL was taken prior to the induction of gRNAs. The induction was carried out by adding anyhydrotetracycline (Abcam Biochemicals #ab145350) to both the growth chamber and the turbidostat media reservoir, in order to achieve a final concentration of 250 ng/ml. Following another six doublings, which took another 9 hours, a post-induction sample of 50 mL was collected.

RNA extractions and barcode library preparations were performed as previously described (Muller et al., 2022, 2020). The cDNA products were amplified with PD780 and RM810 for P(*Z*)-mScarlet libraries, and RM810 and RM511 for P(*TFC1*)-citrine libraries. Sequencing data was first subjected to processing using Cutadapt (Martin, 2011) to trim adapter sequences and deconvolve multiplexed libraries based on embedded nucleotide indices. Trimmed barcodes were then counted as described in (Muller et al., 2020). Barcodes with fewer than 32 counts in at least one of the replicates of the pre-induction samples were excluded from the analysis. The remaining barcodes were evaluated by differential activity analysis using mpralm (Ashuach et al., 2019).

### RT-qPCR

For ZEM RT-qPCR assays, cells were back-diluted from saturation and grown for 9 hours in SCD -URA with 10 nM beta-estradiol and 250 ng/ml anyhydrotetracycline (Abcam Biochemicals #ab145350) when applicable. For eIF4E depletion RT-qPCR assays, cells were back-diluted from saturation and grown for 4 hours in YPD with 500 *μ*M IAA (Sigma-Aldrich #12886) or DMSO. All cells were harvested by centrifugation from mid-log phase. RNA was extract using either acid phenol extraction (Ares, 2012) or Direct-zol RNA miniprep kit (Zymo Research #R2053). Samples extracted with Direct-zol RNA miniprep kit were disrupted by vortexing with glass beads (Sigma-Aldrich #Z250465) for 5 minutes in TRI reagent. RNA samples were treated with DNase I (Thermo Scientific #EN0521) for 1 hour at 37°C, followed by RNA clean up with RNA Clean & Concentrator kit (Zymo Research R1015).

Complementary DNA was synthesized using the SuperScript II reverse transcriptase (Invitrogen #18064014) and random primers according to the manufacturer’s protocol. Quantitative PCR was conducted using the DyNAmo HS SYBR Green kit (Thermo Scientific #F410L) and the primers specified in Table S1. The procedure was carried out on an Mx3000P machine (Stratagene, La Jolla, CA), and standard curves were created using a tenfold dilution sequence of one of the samples that had been prepared.

### β-estradiol titrations

Cells were back-diluted from saturation and grown in SCD -URA with various β-estradiol (Sigma-Aldrich #E8875) concentrations for 8 hours to mid-log phase. Cells were fixed in 4% PFA for 15 minutes, washed and resuspended in PBS + 2 mM EDTA. Fluorescence was measured a BD LSR Fortessa X20 with excitation by the 561 yellow-green laser and captured on the PE-TexasRed channel.

### Growth assays

Cells were grown to mid-log phase (typically 5 mL cultures at OD_600_ 0.6 – 1.0 in YPD unless otherwise indicated), and back-diluted to OD_600_ 0.025 in triplicate. Absorbance measurements at 595nm were measured every 15 min using a 96-well plate reader (Tecan SPARK Multimode Microplate Reader). Growth rates were fit using the R package ‘growthcurver’ (Sprouffske and Wagner, 2016).

## Supplementary Material

1

## Data Availability

All sequencing files are available on NCBI GEO database (accession number GSE231759).
