# Supplementary material for: Dysregulation of amino acid metabolism upon rapid depletion of cap-binding protein eIF4E
Source: bioRxiv. 2023 May 12:2023.05.11.540079. Preprint. [Version 1] doi: 10.1101/2023.05.11.540079 (PMC10197679; doi:10.1101/2023.05.11.540079)
Supplement: 1 [file NIHPP2023.05.11.540079V1-supplement-1.pdf]

bioRxiv preprint doi: https://doi.org/10.1101/2023.05.11.540079; this version posted May 12, 2023. The copyright holder for this preprint (which was not certified by peer review) is the author/funder, who has granted bioRxiv a license to display the preprint in perpetuity. It is made available under aCC-BY 4.0 International license.

Figure S1

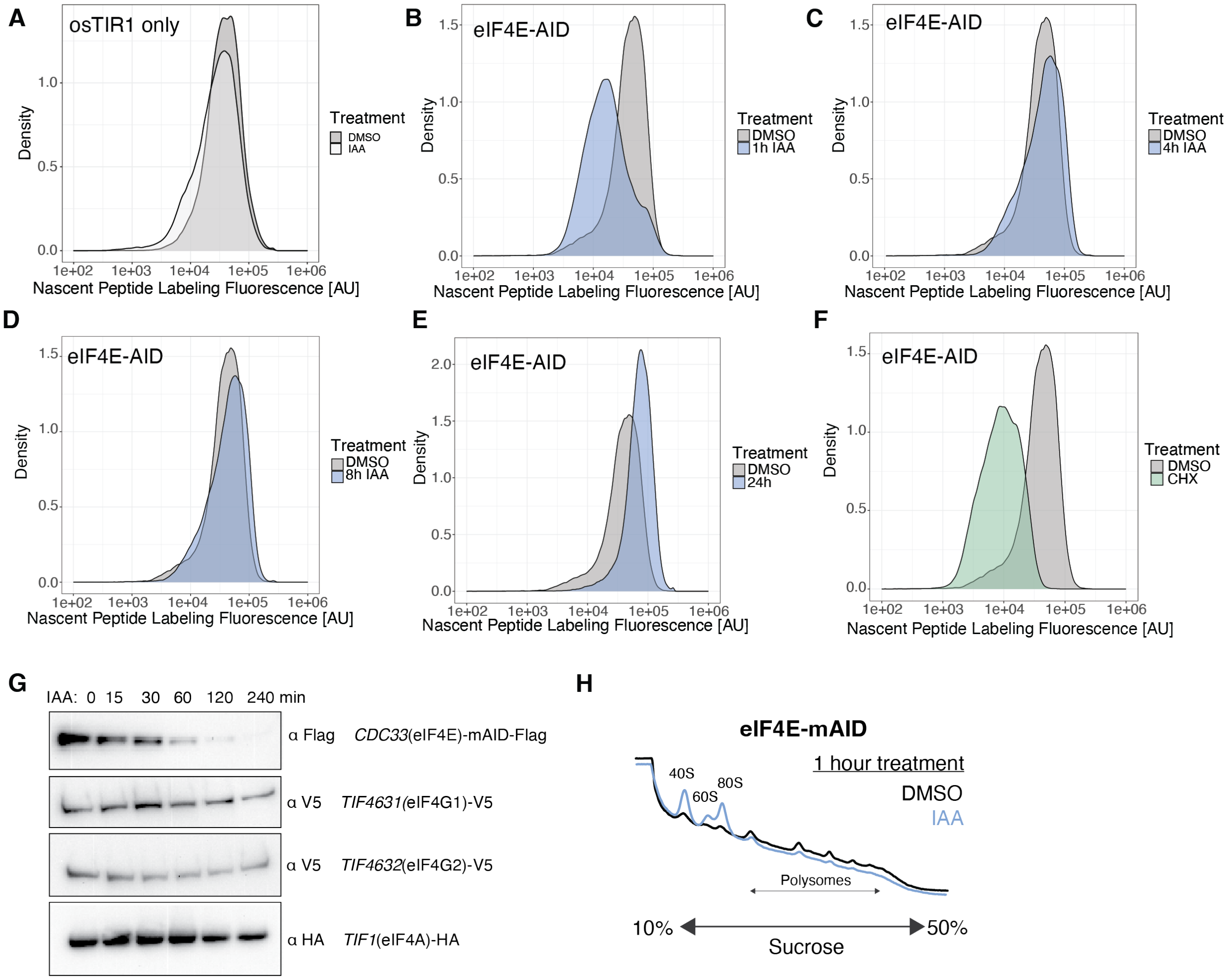

Figure S1: Impact of eIF4E depletion on bulk protein synthesis. (A) Bulk translation measured by nascent peptide metabolic labeling in *osTIR1* only expressing cells. Indole-3-acetic acid (IAA) or DMSO was added for 1 hour and maintained during a 2-hour labeling period with L-Homopropargylglycine (HPG). Fluorescence intensity of Alexa Fluor™ 488 (HPG) signal was measured with flow cytometry. **(B)** Same as in (A) for eIF4E-AID cells. **(C)** Same as in (B) except IAA was added for 4 hours before labeling period. **(D)** Same as in (B) except IAA was added for 8 hours before labeling period. **(E)** Same as in (B) except IAA was added for 24 hours before labeling period. **(F)** Same as in (A) except cycloheximide (CHX) was added concurrently with HPG. **(G)** Western blot analysis for translation initiation expression levels over the course of eIF4E-AID-flag depletion. **(H)** Polysome profiles eIF4E-AID cells treated with IAA or DMSO for 1 hour.

bioRxiv preprint doi: https://doi.org/10.1101/2023.05.11.540079; this version posted May 12, 2023. The copyright holder for this preprint (which was not certified by peer review) is the author/funder, who has granted bioRxiv a license to display the preprint in perpetuity. It is made available under aCC-BY 4.0 International license.

Figure S2

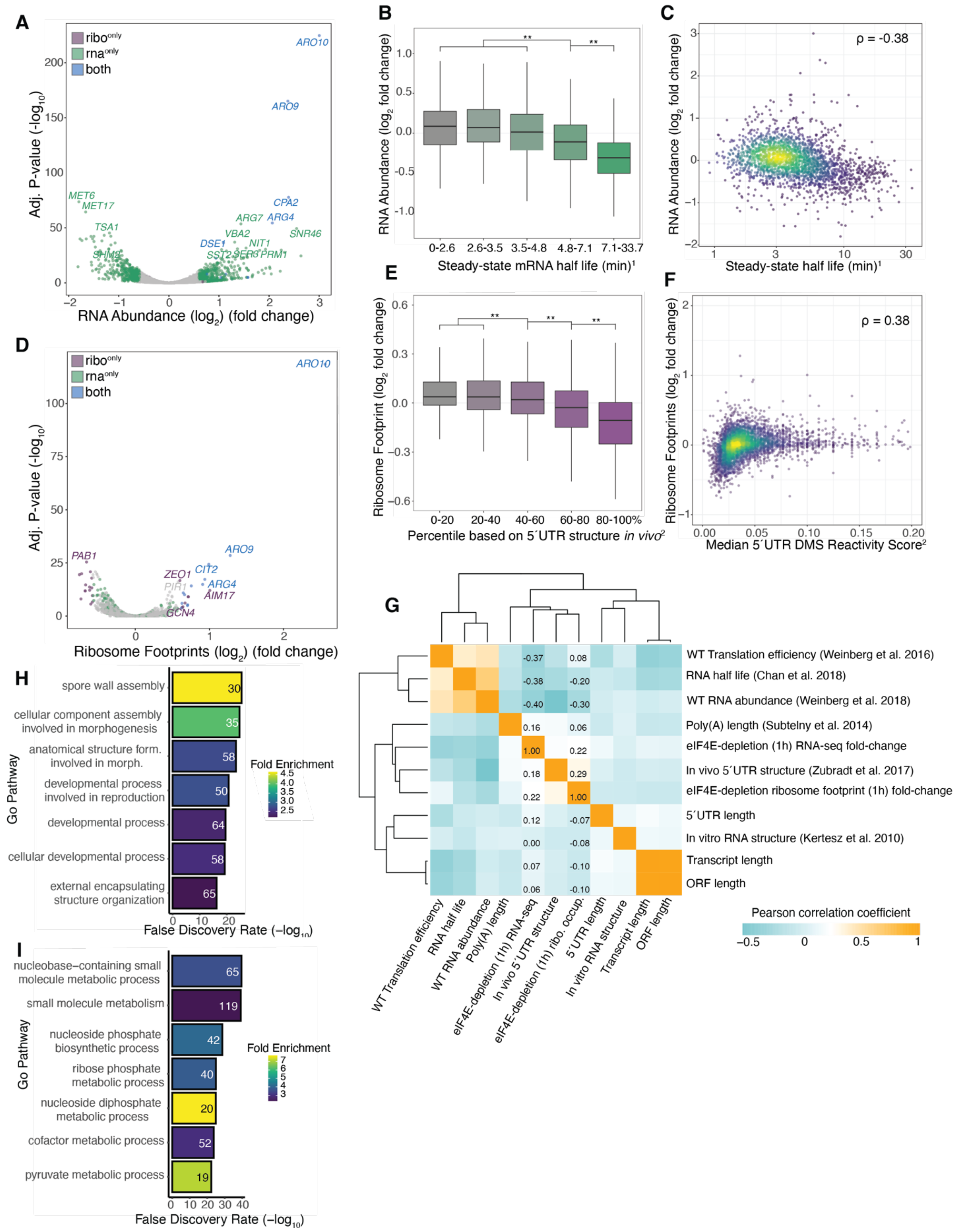

bioRxiv preprint doi: https://doi.org/10.1101/2023.05.11.540079; this version posted May 12, 2023. The copyright holder for this preprint (which was not certified by peer review) is the author/funder, who has granted bioRxiv a license to display the preprint in perpetuity. It is made available under aCC-BY 4.0 International license.

Figure S2: Transcript-level sensitivities after 1 hour of eIF4E depletion. (A) Differential expression after 1 hour of eIF4E depletion measured by RNA-seq. IAA-treated cells are compared with DMSO-treated controls. Color represents significant (adjusted p-value < 0.05) and substantial (absolute fold-change ($\log_2$) > 0.58) changes for RNA-seq and ribosome profiling measurements. **(B)** Box plots of RNA abundance fold change ($\log_2$) after 1 hour of eIF4E depletion for transcripts grouped based on steady-state mRNA half-life (Chan et al., 2018). (**) indicates $p < 0.05$; one-way ANOVA test followed by Tukey's HSD test. **(C)** Scatterplot of steady-state mRNA half life (Chan et al., 2018) and fold change ($\log_2$) after 1 hour of eIF4E depletion measured by RNA-seq. Color represents point density. Correlation coefficient (Spearman's) calculated between $\log_2$ fold changes of RNA abundance and half-life (min). **(D)** Same as (A) except changes in ribosome occupancy measured by ribosome profiling. **(E)** Box plots of ribosome occupancy fold change ($\log_2$) after 1 hour of eIF4E depletion for transcripts grouped based on *in vivo* 5´UTR structure (median DMS reactivity) from DMS-MaPseq data (Zubradt et al., 2017). (**) indicates $p < 0.05$; one-way ANOVA test followed by Tukey's HSD test. **(F)** Scatterplot of DMS reactivity scores over the 5´UTR of transcripts based on DMS-MaPseq data (Zubradt et al., 2017) and fold change ($\log_2$) after 1 hour of eIF4E depletion measured by ribosome profiling. Color represents point density. Correlation coefficient (Spearman's) calculated between $\log_2$ fold changes of RPFs and DMS reactivity score. **(G)** Spearman's correlation coefficients of pairwise comparisons across mRNA parameters including translation efficiency(Weinberg et al., 2016), stability (Chan et al., 2018), expression, poly(A) length (Subtelny et al., 2014), 5´UTR structure (Kertesz et al., 2010; Zubradt et al., 2017), and transcript length. Datasets were hierarchically clustered based on Euclidian distances. Color represents magnitude of correlation coefficient. **(H)** GO analysis for genes which were significantly (adj. p-value < 0.05) up-regulated following eIF4E-depletion (1-hour treatment) in RNA-seq analysis. The most statistically significant entries were chosen and narrowed based on percentage of overlapping genes. **(I)** Same as in (H) for down-regulated genes.

bioRxiv preprint doi: https://doi.org/10.1101/2023.05.11.540079; this version posted May 12, 2023. The copyright holder for this preprint (which was not certified by peer review) is the author/funder, who has granted bioRxiv a license to display the preprint in perpetuity. It is made available under aCC-BY 4.0 International license.

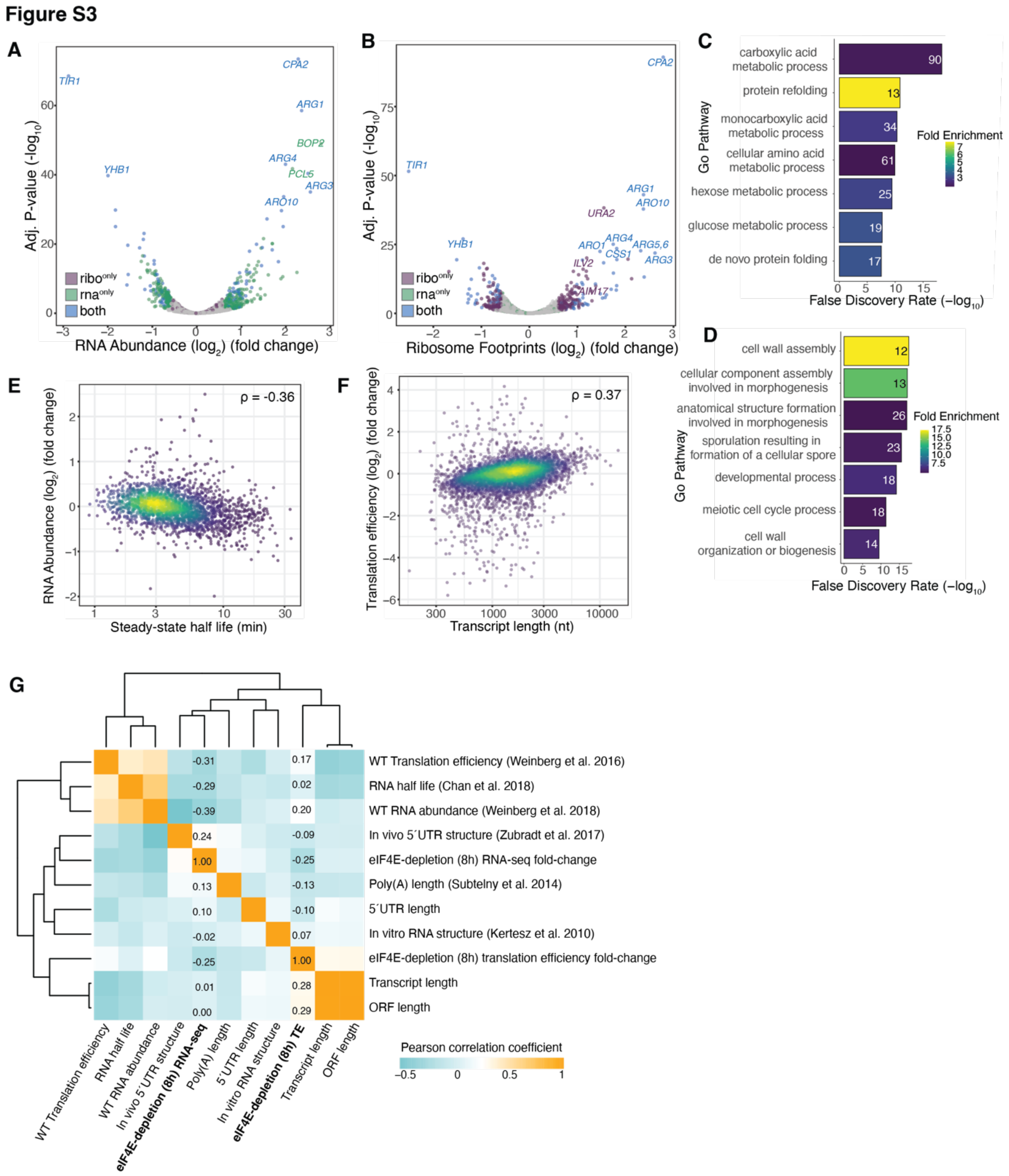

Figure S3: Transcript-level sensitivities after 8 hour of eIF4E depletion. (A) Differential expression after 8 hour of eIF4E depletion measured by RNA-seq. IAA-treated cells are compared with DMSO-treated controls. Color represents significant (adjusted p-value < 0.05) and substantial (absolute fold-change (log_2) > 0.58) changes for RNA-seq and ribosome profiling measurements.

bioRxiv preprint doi: https://doi.org/10.1101/2023.05.11.540079; this version posted May 12, 2023. The copyright holder for this preprint (which was not certified by peer review) is the author/funder, who has granted bioRxiv a license to display the preprint in perpetuity. It is made available under aCC-BY 4.0 International license.

Figure S3: *continued from the previous page***. (B)** Same as (A) except changes in ribosome occupancy measured by ribosome profiling. **(C)** GO analysis for genes which were significantly (adj. p-value < 0.05) up-regulated translation efficiency following eIF4E-depletion (1-hour treatment). The most statistically significant entries were chosen and narrowed based on percentage of overlapping genes. **(D)** Same as in (C) for genes downregulated in translation efficiency. **(E)** Scatterplot of steady-state mRNA half-life (Chan et al., 2018) and fold change (log_2) after 8 hour of eIF4E depletion measured by RNA-seq. Color represents point density. Correlation coefficient (Spearman's) calculated between log_2 fold changes of RNA abundance and half-life (min). **(F)** Scatterplot of transcript length and fold change (log_2) in translation efficiency (TE) after 8 hours of eIF4E depletion. Color represents point density. Correlation coefficient (Spearman's) calculated between log_2 fold changes of TE and transcript length. **(G)** Spearman's correlation coefficients of pairwise comparisons across mRNA parameters including translation efficiency (Weinberg et al., 2016), stability (Chan et al., 2018), expression, poly(A) length (Subtelny et al., 2014), 5´UTR structure (Kertesz et al., 2010; Zubradt et al., 2017), and transcript length. Datasets were hierarchically clustered based on Euclidian distances. Color represents magnitude of correlation coefficient.

bioRxiv preprint doi: https://doi.org/10.1101/2023.05.11.540079; this version posted May 12, 2023. The copyright holder for this preprint (which was not certified by peer review) is the author/funder, who has granted bioRxiv a license to display the preprint in perpetuity. It is made available under aCC-BY 4.0 International license.

Figure S4

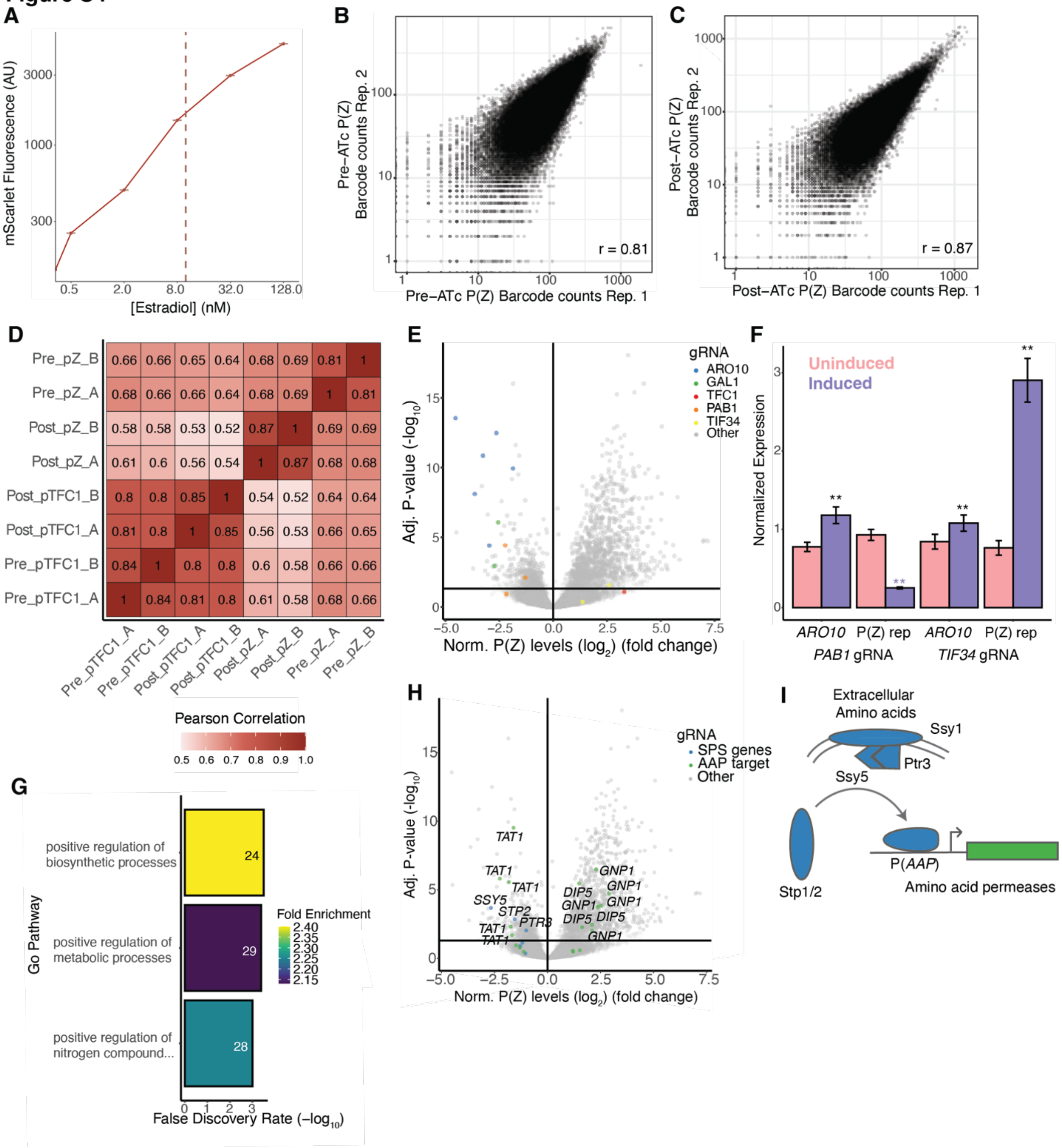

bioRxiv preprint doi: https://doi.org/10.1101/2023.05.11.540079; this version posted May 12, 2023. The copyright holder for this preprint (which was not certified by peer review) is the author/funder, who has granted bioRxiv a license to display the preprint in perpetuity. It is made available under aCC-BY 4.0 International license.

Figure S4: CiBER-seq profile *ARO10* expression regulation. (A)Titration across a range of β-estradiol concentrations in *ARO10-P2A-ZEM* cells to determine a concentration in the linear range where mScarlet fluorescence. Median fluorescence values and standard error are plotted. Line indicates the concentration chosen for CiBER-seq screen. **(B)** Comparison of replicate P(Z) barcode codes from pre-gRNA induction samples and corresponding correlation coefficient (Pearson's). These were chosen as a representative example of replicate samples. **(C)** Same as in (B) for post-gRNA induction samples. **(D)** Pearson's correlation coefficients of pairwise comparisons across all RNA-sequencing samples. Color represents magnitude of correlation coefficient. **(E)** CiBER-seq results showing fold-change (log_2) in P(Z) reporter abundance, relative to P(*TFC1*) reporter levels, for each gRNA. Line indicates significance cutoff (adjusted p-value < 0.05). Color represents gene identity. **(F)** RT-qPCR of *ARO10* and mScarlet reporter expression following *PAB1* or *TIF34* gRNA induction. (**) represents $p < 0.05$ calculated by Student's t test. **(G)** GO analysis for genes targeted by gRNAs that down-regulated P(Z) expression. gRNAs were filtered for fold-change (log_2) > 1 and adjusted p-value < 0.05. The most statistically significant entries were chosen and narrowed based on percentage of overlapping genes. **(H)** Same as in (E) except highlight components of the SPS amino acid sensing pathway and target amino acid permease genes. **(I)** Schematic of the SPS amino acid sensing pathway.

bioRxiv preprint doi: https://doi.org/10.1101/2023.05.11.540079; this version posted May 12, 2023. The copyright holder for this preprint (which was not certified by peer review) is the author/funder, who has granted bioRxiv a license to display the preprint in perpetuity. It is made available under aCC-BY 4.0 International license.

Figure S5

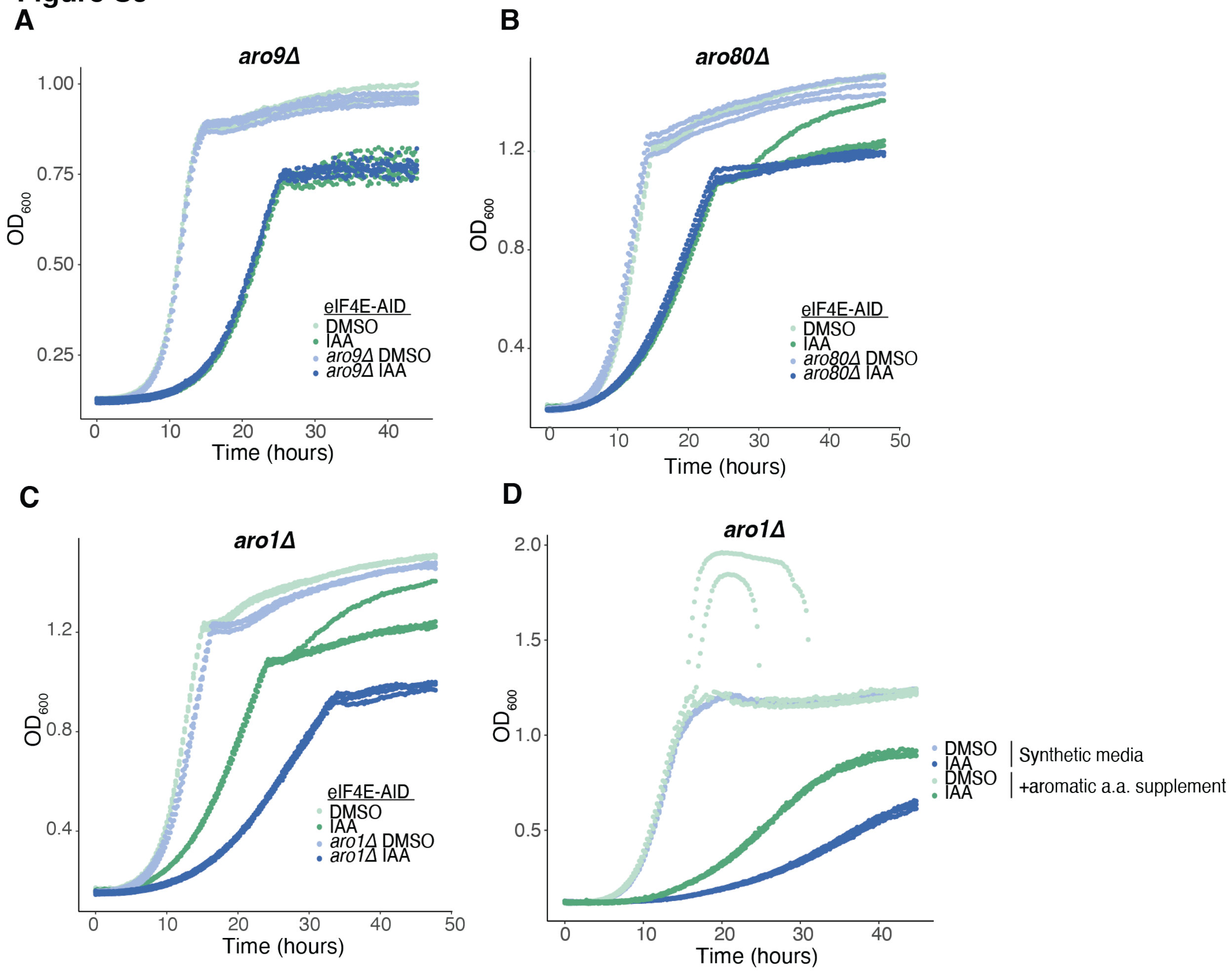

Figure S5: Futile cycles of aromatic amino acid metabolism. (A) Growth curves of eIF4E-AID and eIF4E-AID *aro9Δ* cells maintain in IAA or DMSO. **(B)** Same as in (A) except for *aro80Δ* cells. **(C)** Same as in (A) except for *aro1Δ* cells. **(D)** Growth curves of eIF4E-AID *aro1Δ* cells maintain in IAA or DMSO in synthetic complete media with and without aromatic amino acid supplementation.

bioRxiv preprint doi: https://doi.org/10.1101/2023.05.11.540079; this version posted May 12, 2023. The copyright holder for this preprint (which was not certified by peer review) is the author/funder, who has granted bioRxiv a license to display the preprint in perpetuity. It is made available under aCC-BY 4.0 International license.

Figure S6

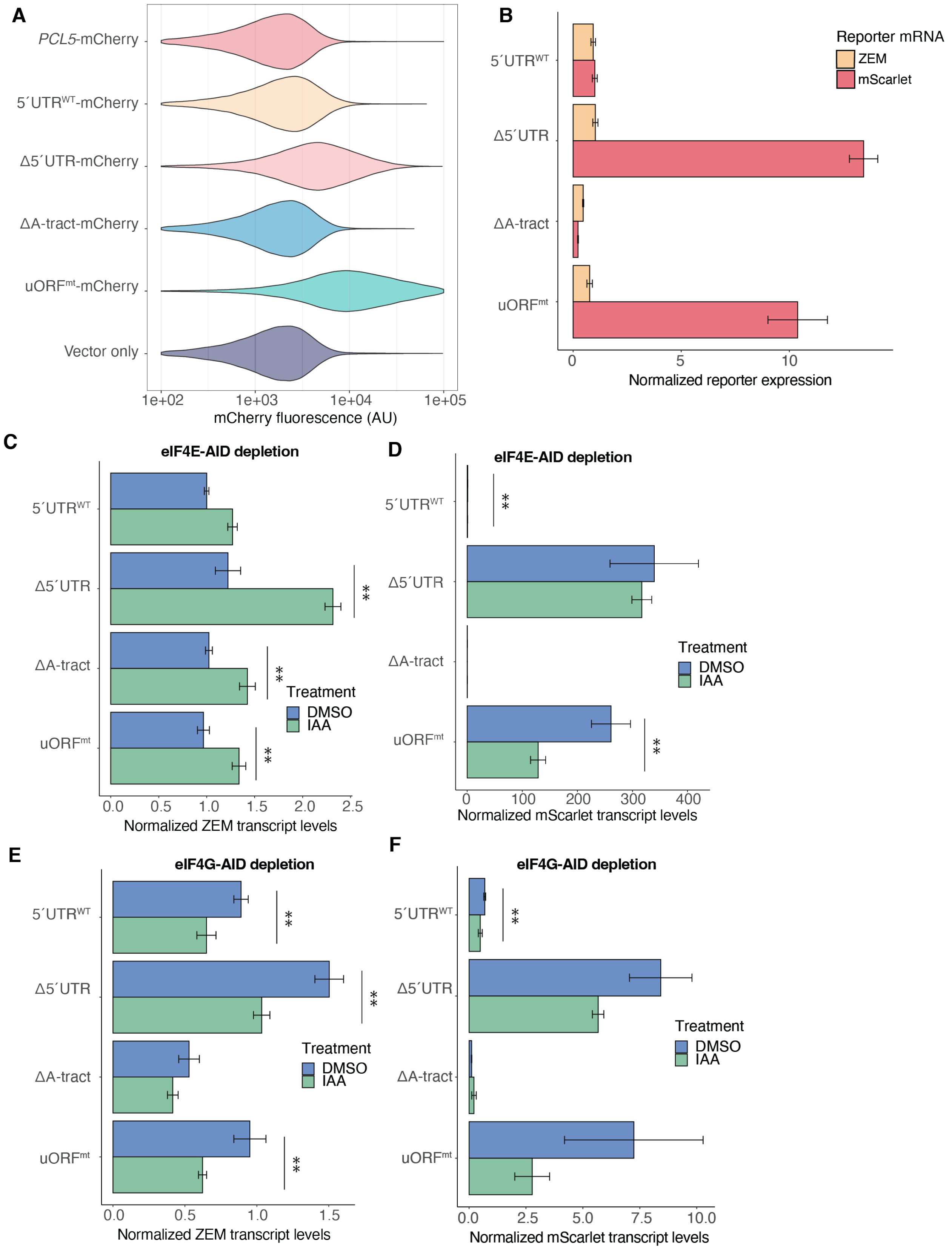

bioRxiv preprint doi: https://doi.org/10.1101/2023.05.11.540079; this version posted May 12, 2023. The copyright holder for this preprint (which was not certified by peer review) is the author/funder, who has granted bioRxiv a license to display the preprint in perpetuity. It is made available under aCC-BY 4.0 International license.

Figure S6: Translation regulation of *PCL5.* (A) Distribution of fluorescence of *PCL5*-mCherry reporters and vector only control. **(B)** RT-qPCR of ZEM transcript and mScarlet reporter expression of *PCL5* 5'UTR. Error bars represent standard deviation, n=3. **(C)** RT-qPCR of ZEM transcript expression of *PCL5* 5'UTR mutants following eIF4E-AID depletion. (**) represents $p < 0.05$ calculated by Student's t test. Error bars represent standard deviation, n=3. **(D)** Same as in (C) except mScarlet reporter transcript. **(E)** Same as in (C) except in eIF4G-AID depletion. **(F)** Same as in (E) except mScarlet reporter transcript.

bioRxiv preprint doi: https://doi.org/10.1101/2023.05.11.540079; this version posted May 12, 2023. The copyright holder for this preprint (which was not certified by peer review) is the author/funder, who has granted bioRxiv a license to display the preprint in perpetuity. It is made available under aCC-BY 4.0 International license.

Figure S7

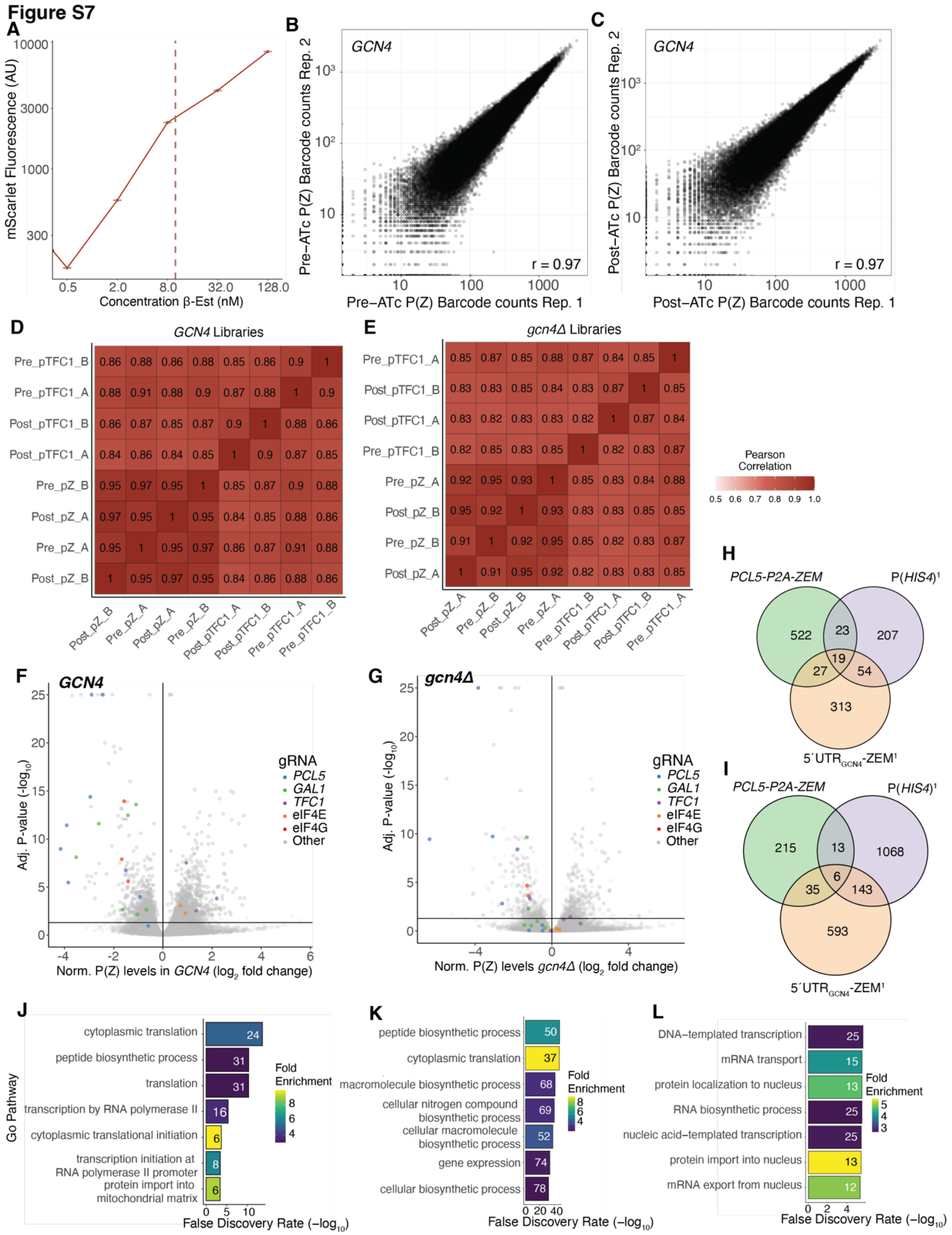

bioRxiv preprint doi: https://doi.org/10.1101/2023.05.11.540079; this version posted May 12, 2023. The copyright holder for this preprint (which was not certified by peer review) is the author/funder, who has granted bioRxiv a license to display the preprint in perpetuity. It is made available under aCC-BY 4.0 International license.

Figure S7: CiBER-seq profile *PCL5* expression regulation. (A) Titration across a range of β-estradiol concentrations in *PCL5-P2A-ZEM* cells to determine a concentration in the linear range where mScarlet fluorescence. Median fluorescence values and standard error are plotted. Line indicates the concentration chosen for CiBER-seq screen. **(B)** Comparison of replicate P(Z) barcode codes from pre-gRNA induction samples and corresponding correlation coefficient (Pearson's). These were chosen as a representative example of replicate samples. **(C)** Same as in (B) for post-gRNA induction samples. **(D)** Pearson's correlation coefficients of pairwise comparisons across all *GCN4* samples. Color represents magnitude of correlation coefficient. **(E)** Same as in (D) except for *gcn4Δ* samples. **(F)** CiBER-seq results showing fold-change (log_2) in P(Z) reporter abundance in *GCN4* cells, relative to P(*TFC1*) reporter levels, for each gRNA. Line indicates significance cutoff (adjusted p-value < 0.05). Color represents gene identity. **(G)** Same as in (F) except in *gcn4Δ* cells. **(H)** Overlap between gRNAs that produced significant (adjusted p-value < 0.05) and substantial increase (fold-change (log_2) > 1) in *PCL5*-P2A-ZEM (this study), P(*HIS4),* and 5´UTR$_{GCN4}$-ZEM reporters expression in previously published CiBER-seq analysis of *GCN4* (Muller et al., 2020)*.* **(I)** Same as in (H) except for gRNAs that produced a substantial decrease in reporter expression. **(J)** GO analysis for genes targeted by gRNAs that up-regulated P(Z) expression in *GCN4* cells. gRNAs were filtered for fold-change (log_2) > 1 and adjusted p-value < 0.05. The most statistically significant entries were chosen and narrowed based on percentage of overlapping genes. **(K)** Same as in (J) for *gcn4Δ* cells. **(L)** Same as in (K) for gRNAs that down-regulated P(Z) expression.

bioRxiv preprint doi: https://doi.org/10.1101/2023.05.11.540079; this version posted May 12, 2023. The copyright holder for this preprint (which was not certified by peer review) is the author/funder, who has granted bioRxiv a license to display the preprint in perpetuity. It is made available under aCC-BY 4.0 International license.

Figure S8

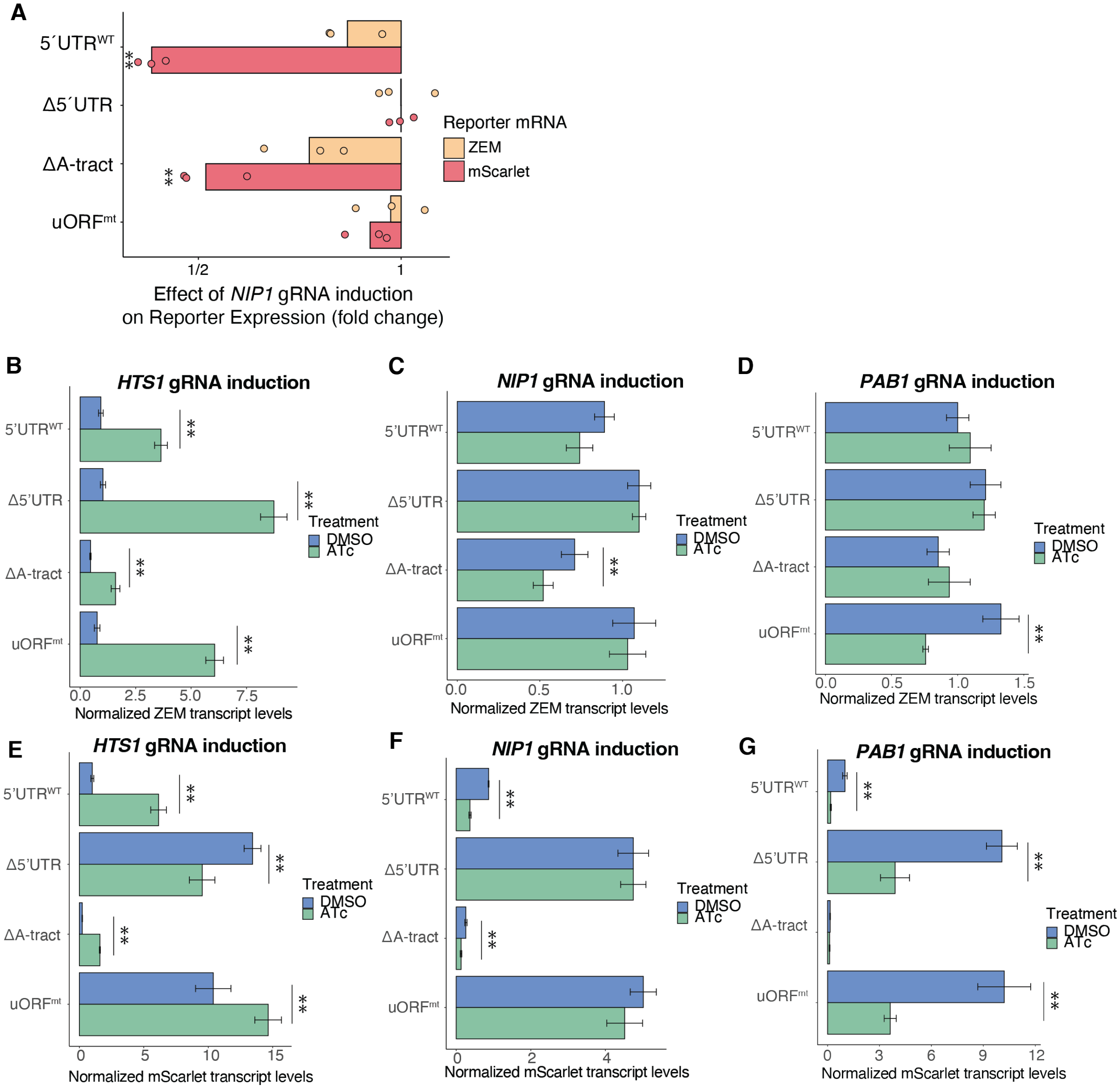

Figure S8: Trans factor regulation of *PCL5* translation. (A) RT-qPCR of ZEM transcript and mScarlet reporter expression of *PCL5* 5´UTR mutants following HTS1 gRNA induction. Individual reporters were normalized to uninduced control. (**) represents p < 0.05 calculated by Student's t test. **(B)** RT-qPCR of ZEM transcript expression of *PCL5* 5'UTR mutants following induction of *HTS1* gRNA. (**) represents p < 0.05 calculated by Student's t test. Error bars represent standard deviation, n=3. **(C)** Same as in (B) except following *NIP1* gRNA induction. **(D)** Same as in (B) except following *PAB1* gRNA induction. **(E)** RT-qPCR of mScarlet transcript expression of *PCL5* 5'UTR mutants following induction of *HTS1* gRNA. **(F)** Same as in (E) except following *NIP1* gRNA induction. **(G)** Same as in (E) except following *PAB1* gRNA induction.
